# Modulating the Evolution of Metastable CaO* for the Near‐Theoretical Performance Breakthrough of Ni/CeO_2_‐CaO in Integrated CO_2_ Capture and Methanation

**DOI:** 10.1002/advs.202503086

**Published:** 2025-03-26

**Authors:** Lifei Wei, Rui Han, Gaoqi Han, Han Yan, Mingke Peng, Zhiyong Li, Chunfeng Song, Qingling Liu

**Affiliations:** ^1^ Tianjin Key Lab of Indoor Air Environmental Quality Control School of Environmental Science and Technology Tianjin University Tianjin 300350 China; ^2^ State Key Laboratory of Engines School of Mechanical Engineering Tianjin University Tianjin 300350 China

**Keywords:** CaO adsorbent, CO_2_ capture and conversion, hydrogen spillover, metastable CaO

## Abstract

Integrated CO_2_ capture and utilization technology based on the calcium looping is burgeoning as an economical and viable strategy for achieving Carbon Neutrality. However, the drawback of easy sintering of CaO limits its potential to maximize CO_2_ capture and conversion. Here, a low‐temperature hydrogen spillover decomposition strategy is proposed to synthesize high‐performance CaO‐based dual‐functional material. This strategy significantly shortens the existence time of the transition state CaO*, enabling CaCO_3_ to be converted into CaO more rapidly. Compared with the traditional sol–gel method, the sintering of CaO is more effectively inhibited. Specifically, NiCa‐400 achieves a CO_2_ capture of 17.8 mmol g^−1^ (theoretical value of 17.8 mmol g^−1^), a CH_4_ yield of 17.2 mmol g^−1^ (192% higher than the conventional method), and a CH_4_ selectivity of 97%. In addition, scale‐up experimental studies further demonstrated its practical scalability. Guided by techno‐economic analysis, coupling the proposed strategy with a coal‐fired power plant can reduce energy consumption by 79% and save investment costs by 23% compared with a conventional carbon capture and utilization (CCU). This work bridges the gap between the actual and theoretical properties of traditional calcium‐based dual‐functional materials and provides a new solution for the high‐value utilization of carbonates.

## Graphical abstract

A low‐temperature hydrogen spillover decomposition strategy is proposed to reveal the influence of the CaCO_3_ parent conversion process on the properties of nascent CaO. Under the guidance of the advanced synthesis strategy, the properties of calcium‐based dual‐functional materials are maximally exploited. New insights are provided for cement, construction, and other industries that need to achieve low‐carbon and efficient conversion of carbonates.

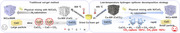

## 1 Introduction

As the demand for energy and resources in human society continues to increase, using large amounts of power has brought severe climate and environmental problems that threaten the continuation of human civilization. Therefore, there is an urgent need to propose transformative solutions to achieve net‐zero CO_2_ emissions and meet the balance between human needs and sustainable development. Carbon capture, utilization, and storage (CCUS) technology is currently the primary way to reduce carbon emissions. However, the intermittent character of its capture and conversion increases the cost of the process and also triggers new carbon emission problems.^[^ ^1^ ^]^ Therefore, under the background of green manufacturing, in 2015, Farrauto et al.^[^ ^2^ ^]^ proposed the concept of dual‐functional materials (DFMs), which coupled CO_2_ capture and conversion at the same temperature and in the same reactor, forming the integrated CO_2_ capture and utilization (ICCU) technology. Thanks to well‐established natural gas transportation facilities, the synthesis of natural gas from CO_2_ and electrolytic hydrogen production can effectively solve the problem of the imbalance between energy supply and demand in time and space.^[^ ^3^ ^]^ Therefore, integrated CO_2_ capture and methanation (ICCM) has become a carbon reduction solution with long‐term development potential.

DFMs applied to ICCM are mainly composed of loaded metal catalysts (e.g., Ru,^[^ ^4^ ^]^ Ni,^[^ ^5^ ^]^ Rh,^[^ ^6^ ^]^ Pd,^[^ ^7^ ^]^ Co,^[^ ^8^ ^]^ Pt,^[^ ^9^ ^]^ etc.) and adsorbents (e.g., CaO,^[^ ^10^ ^]^ Li_4_SiO_4_,^[^ ^11^ ^]^ MgO,^[^ ^12^ ^]^ K_2_O,^[^ ^13^ ^]^ Na_2_O,^[^ ^14^ ^]^ etc.). Among the catalysts, Ni‐based catalysts loaded on CeO_2_ containing abundant oxygen vacancies have attracted much attention due to their low cost and excellent performance. Sun et al.^[^ ^15^ ^]^ achieved 85.8% CH_4_ selectivity using a catalyst of 0.5 wt.% Ni/CeO_2_. Typically, the catalyst determines the hydrogenation activity and selectivity of the product, while the adsorbent is inextricably linked to the yield of the product.^[^ ^16^ ^]^ Among the adsorbents, CaO is widely used in ICCM because of its excellent capture performance and low cost. Meanwhile, since the decomposition temperature of CaCO_3_ (the carbonation product of CaO) is usually higher than the reaction temperature of methanation (<600 °C), thus methanation can be realized by gas‐solid reaction, which is conducive to reducing CO_2_ escape.^[^ ^17^ ^]^ However, in medium‐temperature methanation reactions (<500 °C), CaO usually exhibits low CO_2_ capture performance, and CaCO_3_ fails to be fully converted to methane.^[^ ^18^ ^]^ Therefore, the actual ICCM performance of CaO‐based DFMs often falls short of the theoretical performance. Moreover, the CaO adsorbent tends to sinter during the cycle, decreasing stability.^[^ ^19^ ^]^ Whether the sintering of calcium looping reactions occurs during carbonation or calcination has always been controversial.^[^ ^20^ ^]^ However, more studies have shown that sintering is more likely to occur during the phase transition and is related to the CO_2_ concentration on the adsorbent surface.^[^ ^21^ ^]^ A crystal structure transformation usually accompanies the process from CaCO_3_ to CaO, and the crystallographic structure is often one of the major influences on the dominant material activity.^[^ ^22^ ^]^

In 1958, Hyatt et al.^[^ ^23^ ^]^ initially proposed that the crystal structure transformation of CaCO_3_ to CaO occurs in two steps. First, CO_2_ desorbs from the CaCO_3_ group, forming an unstable CaO form with a structure intermediate between the rhombic form of CaCO_3_ and the cubic form of CaO, which is also known as metastable calcium oxide (CaO*) (Equation 1). Then, CaO* transforms into the stable CaO structure (Equation 2). Subsequently, the first visual identification of CaO* was obtained in 1974 when Beruto et al.^[^ ^24^ ^]^ found an intermediate layer of ≈30 µm thickness between calcite and calcium oxide by scanning electron microscopy (SEM). Navarro et al.^[^ ^25^ ^]^ explained the formation of CaO*. They suggest that the evolution of CaCO_3_ to CaO follows a topological orientation mechanism, where the loss of CO_2_ in the CaCO_3_ groups leads to an accumulation of strain, and CO_2_ desorption as a result of the stress release leads to the formation of a crystal structure with cavities (i.e., CaO*). Directional aggregation and sintering of CaO* then occur, culminating in the formation of CaO. This indicates that the presence of CaO* is closely related to the grain size of CaO. Valverde et al.^[^ ^26^ ^]^ investigated the role of CaO* at near equilibrium. They noted that the aggregation of CaO* nanocrystals usually controls the growth of CaO grain size, and thus, hindering the aggregation of CaO* nanocrystals would be beneficial in obtaining smaller‐sized CaO. In addition, Beruto et al.^[^ ^27^ ^]^ found that CaCO_3_ decomposition in a vacuum produced CaO with a higher specific surface area and higher reactivity properties. Meanwhile, Sarrion et al.^[^ ^28^ ^]^ found that lowering the partial pressure of CO_2_ during calcination helped to reduce the strength of CO_2_ adsorption on the surface of CaO, weakening the strength of van der Waals gravity between CaO* nanocrystals and achieving the effect of hindering CaO* aggregation and obtaining smaller‐sized CaO. Therefore, reducing the CO_2_ concentration during CaCO_3_ conversion can hinder the aggregation of CaO, which is essential for obtaining more active CaO‐based DFMs. However, vacuum and low partial pressure of CO_2_ are unsuitable for industrial production, so it is necessary to propose a simpler and more efficient way to realize the regulation of CaO*.

(1) $$\mathrm{CaC}O_{3}↔{\mathrm{CaO}}^{*}+CO_{2}$$

(2) $${\mathrm{CaO}}^{*}↔\mathrm{CaO}$$

Here, we report the synthesis of a highly active DFMs (NiCa‐400R) using a low‐temperature hydrogen spillover decomposition strategy, which effectively hinders the aggregation of CaO. Compared with the conventional scheme of doping by inert carriers to improve the cycling performance of CaO, our proposed strategy operates more simply and consumes less energy. More surprisingly, the properties of the obtained NiCa‐400R achieve a breakthrough in the near‐theoretical stoichiometric ratio. Combined with in situ characterization, we find that hydrogen‐rich calcination conditions favor the evolution of parent CaCO_3_ into smaller‐sized nascent CaO, which has a “genetic effect”.

## 2 Results and Discussion

### 2.1 Structural Properties of NiCa‐T

Thermal weight loss analysis was performed on the dried gel to investigate their decomposition at different temperatures (Figure S1, Supporting Information). The weight loss of the dried gel was divided into three parts, with Ca_3_(C_6_H_5_O_7_)_2_·4H_2_O dehydration and C_6_H_8_O_7_ decomposition occurring at temperatures below 170 °C (L1). When the temperature reaches 400 °C, Ca_3_(C_6_H_5_O_7_)_2_ decomposition and product combustion occur to produce CaCO_3_ (L2). When the temperature reaches ≈680 °C, CaCO_3_ decomposes, and CaO is obtained (L3). Powder X‐ray diffraction (XRD) patterns further confirmed the presence of CaCO_3_ in Ca‐400, Ca‐500, and Ca‐600, and no CaO was observed. CaCO_3_ disappeared in Ca‐700 and Ca‐800 and existed as CaO (Figure S2, Supporting Information).

**Figure 1:**
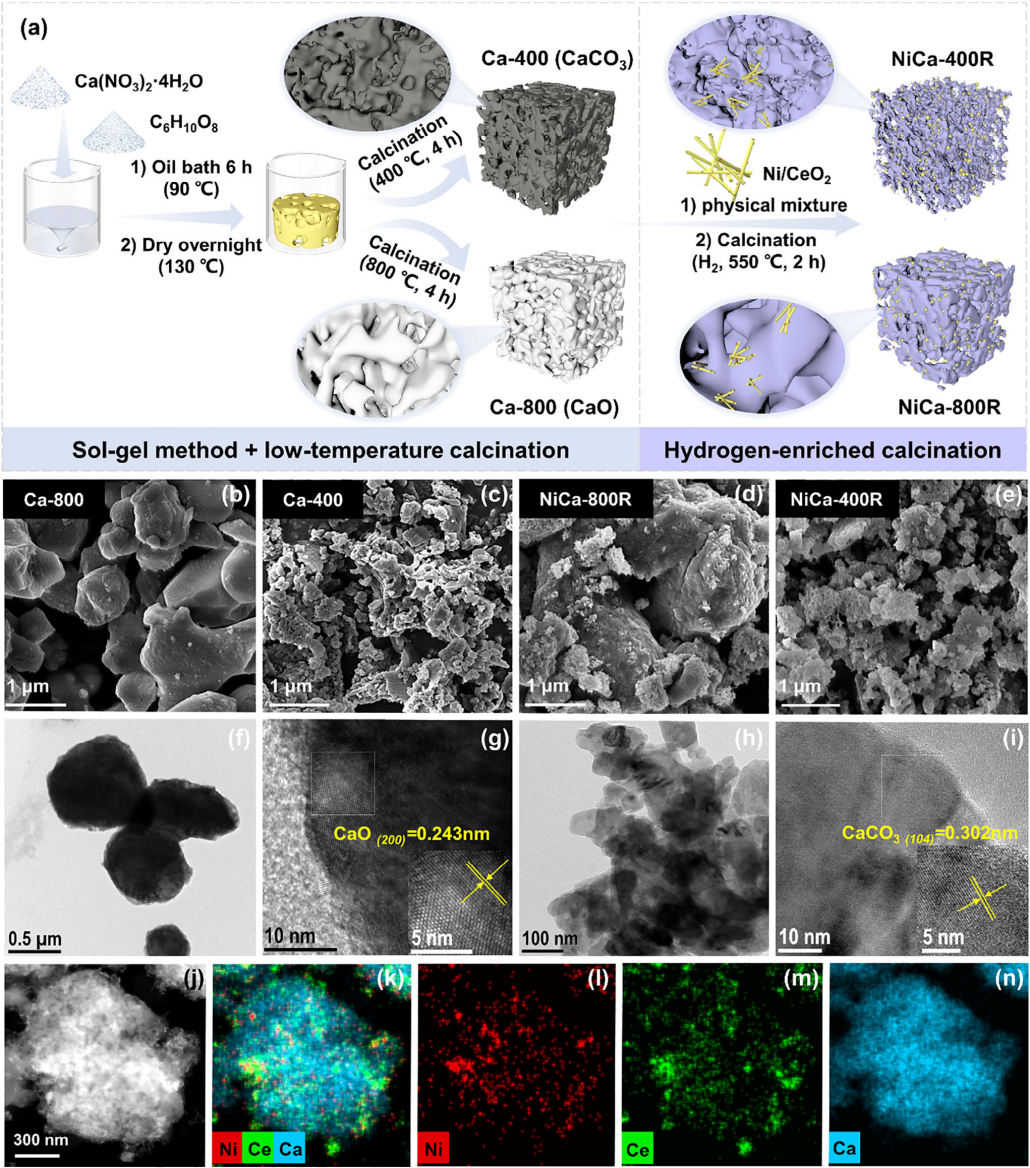
Schematic diagram of low‐temperature hydrogen spillover strategy (a), SEM images of Ca‐800 (b), Ca‐400 (c), NiCa‐800R (d), and NiCa‐400R (e). TEM and high‐resolution TEM of Ca‐800 (f,g), Ca‐400 (h,i). STEM (j) and EDX of NiCa‐400R (k–n).

Based on the effect of different calcination temperatures on the morphology of Ca species present, we propose a low‐temperature hydrogen spillover strategy based on the sol‐gel method (**Figure** **1**a). The sintering of CaCO_3_ (Taman temperature ≈533 °C) was inhibited by low‐temperature calcination (400 °C) first. Subsequently, the hydrogen spillover effect generated by Ni/CeO_2_ caused CaCO_3_ to evolve into CaO. From the SEM images, CaO (Ca‐800) obtained by high‐temperature calcination showed a blocky morphology (Figure 1b). Whereas CaCO_3_ (Ca‐400) obtained by calcination at 400 °C exhibited a rich coral‐like pore structure (Figure 1c). After CaCO_3_ decomposition by hydrogen spillover, the resulting NiCa‐400R had a richer pore structure than that of NiCa‐800R (Figure 1d,e). In addition, transmission electron microscope (TEM) results showed that Ca‐400 has a smaller grain size (Figure 1f,h). Lattice streak analysis confirmed the compositions of Ca‐800 and Ca‐400 as CaO and CaCO_3_, respectively (Figure 1g,i). The reduced Ni/CeO_2_R showed a perfect rod‐like structure with black particles rich in Ni (Figure S3a, Supporting Information). Since Ni is highly oxidizable, it exists in the form of NiO (Figure S3b, Supporting Information). The lattice streak analysis and XRD patterns of NiCa‐400R both indicate that CaCO_3_ is converted into CaO after prereduction (Figures S3d and S10, Supporting Information). The energy dispersive X‐ray elemental mapping (EDX) results showed that the surface of the CaO particles was uniformly covered by a layer of catalyst and both were well‐mixed on the nanoscale (Figure 1j–n).

N_2_ adsorption‐desorption isotherms and pore size distributions confirmed that high‐temperature calcination accelerated the sintering of the calcium‐based materials (Figure S4, Supporting Information). The Brunauer–Emmet–Teller (BET) surface area of Ca‐400 calcined at low temperature is more than ten times that of Ca‐800 calcined at high temperature. Due to the higher specific surface area of Ni/CeO_2_, the specific surface areas of physically mixed NiCa‐400R and NiCa‐800R have increased significantly compared with Ca‐400 and Ca‐800 respectively. It is worth noting that after decomposition by hydrogen spillover, the BET surface area of NiCa‐400R is still higher than that of NiCa‐800R (Table S1, Supporting Information). The pore size distributions of BJH indicated that the adsorbents and DFMs prepared at different calcination temperatures were mesoporous in structure, with NiCa‐400R having the largest pore volume.

### 2.2 Effect of Calcination and Reaction Temperatures

The ICCM properties of NiCa‐T synthesized at different calcination temperatures are shown in Figure S5a (Supporting Information). The CO_2_ capture decreased from 17.6 to 11.38 mmol g^−1^, and the CH_4_ yield decreased from 16.2 to 8.9 mmol g^−1^ as the calcination temperature increased from 400 to 800 °C. Notably, when the initial state of the adsorbent was CaCO_3_ (T ≤ 600 °C), the DFMs exhibited activities close to the theoretical values. However, when the initial state of the adsorbent was CaO (T ≥ 700 °C), the activity of the materials decreased significantly. Considering the better cyclic stability and lower synthesis energy consumption of NiCa‐400 (Figure S5, Supporting Information), it was selected to be discussed in detail in the following section, compared to NiCa‐800, which has the highest degree of sintering.

The reaction temperature generally plays a crucial role in the reaction performance. Figure S6a,b (Supporting Information) respectively displays the reaction performance of NiCa‐400 and NiCa‐800 at 350, 450, and 550 °C. At low temperatures, both the rate of CO_2_ capture by CaO and the rate of the hydrogenation reaction of CaCO_3_ are relatively slow. As a result, the reaction performance of both NiCa‐400 and NiCa‐800 samples shows a gradual upward trend with the increase in temperature. However, at the same reaction temperature, the reaction performance of NiCa‐400 is consistently superior to that of NiCa‐800. Given that the catalyst compositions of NiCa‐400 and NiCa‐800 are the same, the outstanding performance of NiCa‐400 can be attributed to its abundant specific surface area. It is worth noting that when the reaction temperature is 350 °C, the CH_4_ yields of NiCa‐400 and NiCa‐800 are much lower than the CO_2_ capacity. This is because the gas‐solid reaction of CaCO_3_ hydrogenation has slow mass transfer at low temperatures.^[^ ^29^ ^]^ After the surface CaCO_3_ has reacted, it is difficult for the CaCO_3_ in the deeper layers to react. The XRD pattern of the post‐reaction DFMs also showed that most CaCO_3_ remained in the material reacted at 350 °C (Figure S6c,d, Supporting Information).

### 2.3 ICCM Performance of the DFMs

**Figure 2:**
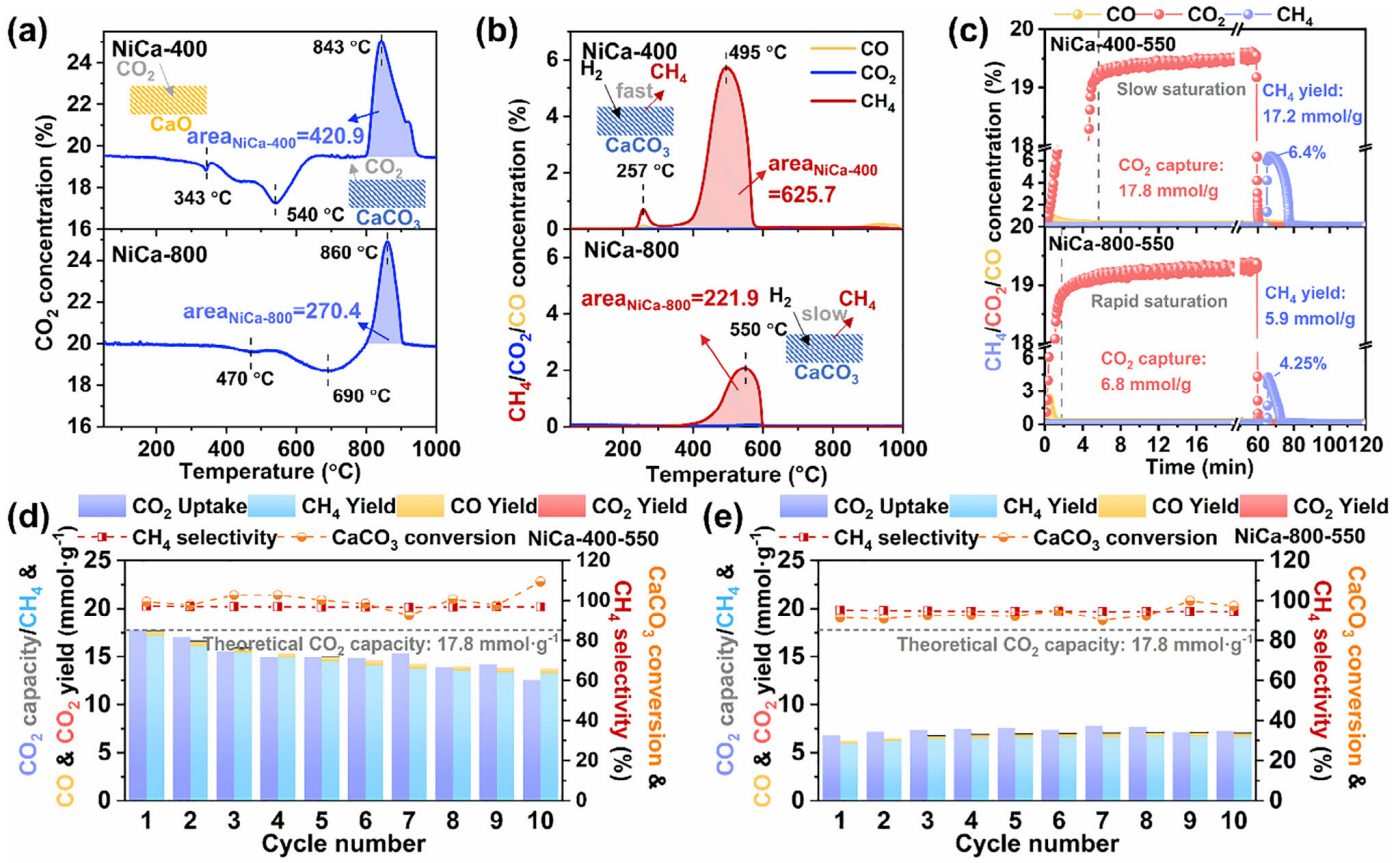
CO_2_‐TPSR (a) and H_2_‐TPSR (b) of NiCa‐400 and NiCa‐800; real‐time concentration variations for the first cycle and 10‐cycle performance of NiCa‐400 (c,d) and NiCa‐800 (c,e) at 550 °C (capture stage: 20% CO_2_/N_2_, 60 min; methanation stage: 100% H_2_/N_2_, 60 min; 100mL min^−1^).

The performance of NiCa‐400 and NiCa‐800 was evaluated more intuitively by temperature‐programmed surface reaction (TPSR). For the CO_2_‐TPSR, NiCa‐400 and NiCa‐800 showed a similar trend of downward CO_2_ capture peaks followed by upward CO_2_ desorption peaks (**Figure** **2**a). In the high‐temperature range, the area of the CO_2_ desorption peak can reflect the CO_2_ capture capacity of different samples. The area of the CO_2_ desorption peak of NiCa‐400 is significantly larger than that of NiCa‐800, which fully demonstrates the remarkable superiority of NiCa‐400 in terms of CO_2_ capture performance. For the H_2_‐TPSR, NiCa‐400 first exhibits a CH_4_ peak at ≈257 °C. Subsequently, the area of the CH_4_ generation peak near 495 °C far exceeds that of NiCa‐800, which further indicates that the methanation performance of NiCa‐400 is equally outstanding (Figure 2b).

The CO_2_‐programmed warming desorption (CO_2_‐TPD) results showed that NiCa‐400R and NiCa‐800R exhibited four major desorption peaks (Figure S7, Supporting Information). Among them, the CO_2_^I^ and CO_2_^II^ peaks were attributed to the weakly basic sites. CO_2_^III^ with peak centers at 500∼650 °C was attributed to moderately basic sites. And CO_2_^IV^ with a peak center higher than 700 °C is classified as a strongly basic site. Since the strong basic sites are mainly caused by the decomposition of carbonate, which is much higher than the reaction temperature of methanation, the relevant ones in the ICCM process should be the moderately basic sites.^[^ ^30^ ^]^ NiCa‐400R has more substantial desorption peaks of CO_2_^III^, which suggests that NiCa‐400R has a stronger affinity for CO_2_.^[^ ^31^ ^]^

The hydrogen spillover intensity was measured using hydrogen‐programmed warming desorption (H_2_‐TPD) (Figure S8, Supporting Information). There were two peaks in the range of 100∼600 °C. Peak I, which occurs at low temperatures, is attributed to the chemisorption of hydrogen by Ni, while peak II, which occurs at high temperatures, is attributed to hydrogen spillover.^[^ ^32^ ^]^ For NiCeO_2_R, peak II is generated by hydrogen spillover to CeO_2_ after Ni cleavage. For NiCeO_2_R‐Ca400, peak II was caused by hydrogen spillover from Ni to CeO_2_, hydrogen spillover to the adsorbent, and hydrogen spillover to the adsorbent via the support.^[^ ^33^ ^]^ The amount of hydrogen spillover for NiCeO_2_R‐Ca400 was significantly larger than that for NiCeO_2_R, suggesting that the addition of Ca‐400 was beneficial in driving the hydrogen activation and spillover phenomena, which in turn was beneficial for the positive methanation process.^[^ ^34^ ^]^

The cycling performance of NiCa‐400 and NiCa‐800 was then systematically tested through a continuous ICCM process. Figure 2c shows the variation of various gas concentrations during the first cycle. NiCa‐400 and NiCa‐800 first undergo a period of CO_2_ adsorption and subsequently reach saturation. Remarkably, in the capture stage, NiCa‐400 took longer to reach CO_2_ saturation. In the methanation stage, the concentration and rate of CH_4_ production were significantly higher than that of NiCa‐800. After 10 ICCM cycles, the CO_2_ capture performance of NiCa‐400 decreased from 17.8 to 12.5 mmol g^−1^, the CH_4_ yield decreased from 17.2 to 13.2 mmol g^−1^, and the CH_4_ selectivity was maintained above 97% (Figure 2d). The CO_2_ capture of NiCa‐800 was consistently ≈7.4 mmol g^−1^, and the CH_4_ yield was consistently around 6.5 mmol/g, which is less than half of NiCa‐400 (Figure 2e).

After 10 cycles, sintering occurred in both NiCa‐400 and NiCa‐800. However, compared with NiCa‐800, the pore structure of NiCa‐400 remained more abundant (Figure S9, Supporting Information). The BET surface area of NiCa‐800 decreased by 10.7% and that of NiCa‐400 by 33.7% after cycling (Table S1, Supporting Information). In addition, after cycling, the grain size of CaO in NiCa‐400 has increased nearly two‐fold. This is because the temperature under the reaction conditions is higher than the Tamman temperature of CaCO_3_ (533 °C), making sintering inevitable during the reaction. As for NiCa‐800, the size of CaO in it has only increased slightly. This is because severe sintering occurred during the synthesis of CaO, so the increase in the size of CaO after cycling is not significant. It is worth noting that although the sintering of NiCa‐400 is more pronounced during the reaction, the grain size of CaO in NiCa‐400 is still smaller than that in NiCa‐800 after the reaction. Furthermore, we also noticed that after cycling, the size of CeO_2_ in both systems increased slightly, indicating that the catalyst also underwent sintering during the reaction. However, given that the CaCO_3_ conversion in both systems is higher than 90% throughout the cycling process, this shows that the slight sintering of the catalyst has a minimal impact on the reaction.

### 2.4 Crystal Phase Evolution of the DFMs

**Figure 3:**
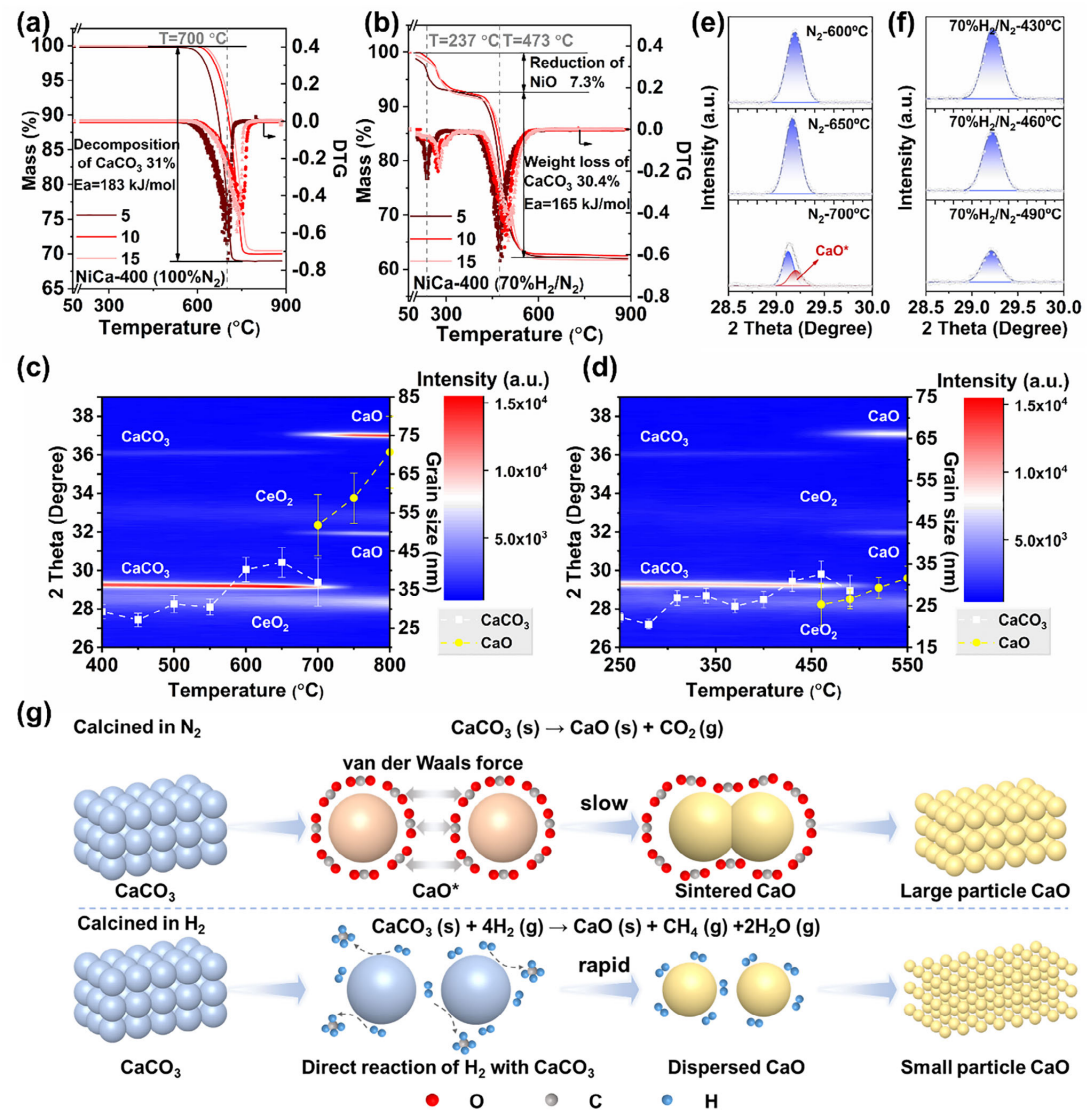
Thermal weight loss and DTG curves of NiCa‐400 at different heating rates (5,10,15 °C min^−1^) in N_2_ (a) and 70% H_2_/N_2_ (b). In situ XRD patterns and grain size changes of NiCa‐400 in N_2_ (c) and 70% H_2_/N_2_ (d). Results of peak fitting during crystalline phase transformation of NiCa‐400 in N_2_ (e) and 70% H_2_/N_2_ (f) for in situ XRD testing. Mechanism of H_2_ spillover promoting CaCO_3_ decomposition (g).

The decomposition properties of NiCa‐400 were probed with a thermogravimetric analyzer under N_2_ and 70% H_2_/N_2_ conditions, respectively (**Figure** **3**a,b). The weight loss near 700 °C under N_2_ conditions was attributed to the decomposition of CaCO_3_. In the presence of H_2_, the first segment of weight loss was attributed to the reduction of NiO, followed by CaCO_3_ weight loss near 473 °C. The activation energy of the weight‐loss section of CaCO_3_ under N_2_ conditions (183 kJ mol^−1^) was higher than that under H_2_ conditions (165 kJ mol^−1^), suggesting that calcination under a hydrogen‐rich atmosphere facilitates the conversion of CaCO_3_ to CaO at lower temperatures (Figure S11 and Table S2, Supporting Information).

To further elucidate the role of the hydrogen‐rich environment in the calcination process, the crystalline phase changes and grain size changes of NiCa‐400 were observed by in situ XRD under N_2_ and 70% H_2_/N_2_ conditions. Under N_2_ conditions, with increasing temperatures, the formation of CaO started at 650 °C, and CaCO_3_ was converted entirely to CaO around 700 °C (Figure 3c). Under hydrogen‐rich conditions, diffraction peaks attributed to CaO were observed near 450 °C, and CaCO_3_ was wholly converted to CaO near 500 °C (Figure 3d). The initial grain size of CaCO_3_ was ≈29.1 nm under N_2_ conditions, and the grain size gradually increased with the temperature rise, indicating that the sintering of CaCO_3_ occurred during the calcination process.^[^ ^21a^ ^]^ After the decomposition of CaCO_3_ to CaO, the grain size of CaO was also significantly increased from the initial 51.8 to 70.7 nm. This was attributed to high‐temperature calcination resulting in the sintering of CaO^[^ ^35^ ^]^ (Figure 3d). Under the 70% H_2_/N_2_ condition, the initial grain size of CaCO_3_ was similar to the N_2_ condition. The grain size of CaCO_3_ was basically maintained below 30 nm with increasing temperature. After conversion to CaO, the grain size of CaO increased slightly, but the final grain size was only 31.6 nm, which was smaller than the initial size of CaO under N_2_ conditions.

Since the diffraction peaks of CaO* are very close in position to those of CaCO_3_, the presence of CaO* can only be further determined by analyzing the experimental peaks.^[^ ^26^ ^]^ Therefore, we investigated the diffraction peaks at the relevant temperature points where the crystalline phase transition occurs under N_2_ and 70% H_2_/N_2_ conditions, respectively (Figure 3e,f). The peaks belonging to CaO* were obtained by fitting at 700 °C under N_2_ conditions. In contrast, no diffraction peaks belonging to CaO* were analyzed under 70% H_2_/N_2_ conditions, indicating that CaO* was present for a short period and that the evolution of CaCO_3_ to CaO occurred rapidly.^[^ ^28^ ^]^

The mechanism of H_2_ spillover promoting CaCO_3_ decomposition can be explained by Figure 3g. Under the N_2_ atmosphere, the desorption of CO_2_ groups from CaCO_3_ resulted in CaO*.^[^ ^25^ ^]^ Meanwhile, the adsorbed CO_2_ on the surface enhances the van der Waals forces between CaO*, promoting the aggregation of CaO* and forming larger‐size CaO.^[^ ^21^, ^28^ ^]^ In contrast, under the H_2_ atmosphere, H_2_ reacts directly with CaCO_3_, inhibiting the presence of CaO* and promoting the rapid evolution of CaCO_3_ into smaller‐sized CaO. In addition, it is noteworthy that CaO and H_2_O co‐exist in the products of the CaCO_3_ hydrogenation reaction. To deeply investigate whether Ca(OH)_2_ might be a reaction product, we carefully compared the XRD pattern of the CaCO_3_ conversion stage under hydrogenation conditions with the Powder Diffraction File (PDF) of Ca(OH)_2_ (Figure S12, Supporting Information). The results showed that no diffraction peaks attributed to Ca(OH)_2_ were observed in the profiles. Based on the temperature conditions, the temperature of CaCO_3_ to CaO conversion is near 450 °C, which is beyond the temperature at which Ca(OH)_2_ begins to decompose (372 °C, Figure S13, Supporting Information). Therefore, the product of CaCO_3_ hydrogenation was confirmed to be CaO, and this conclusion is in agreement with the previous research results.^[^ ^36^ ^]^

**Figure 4:**
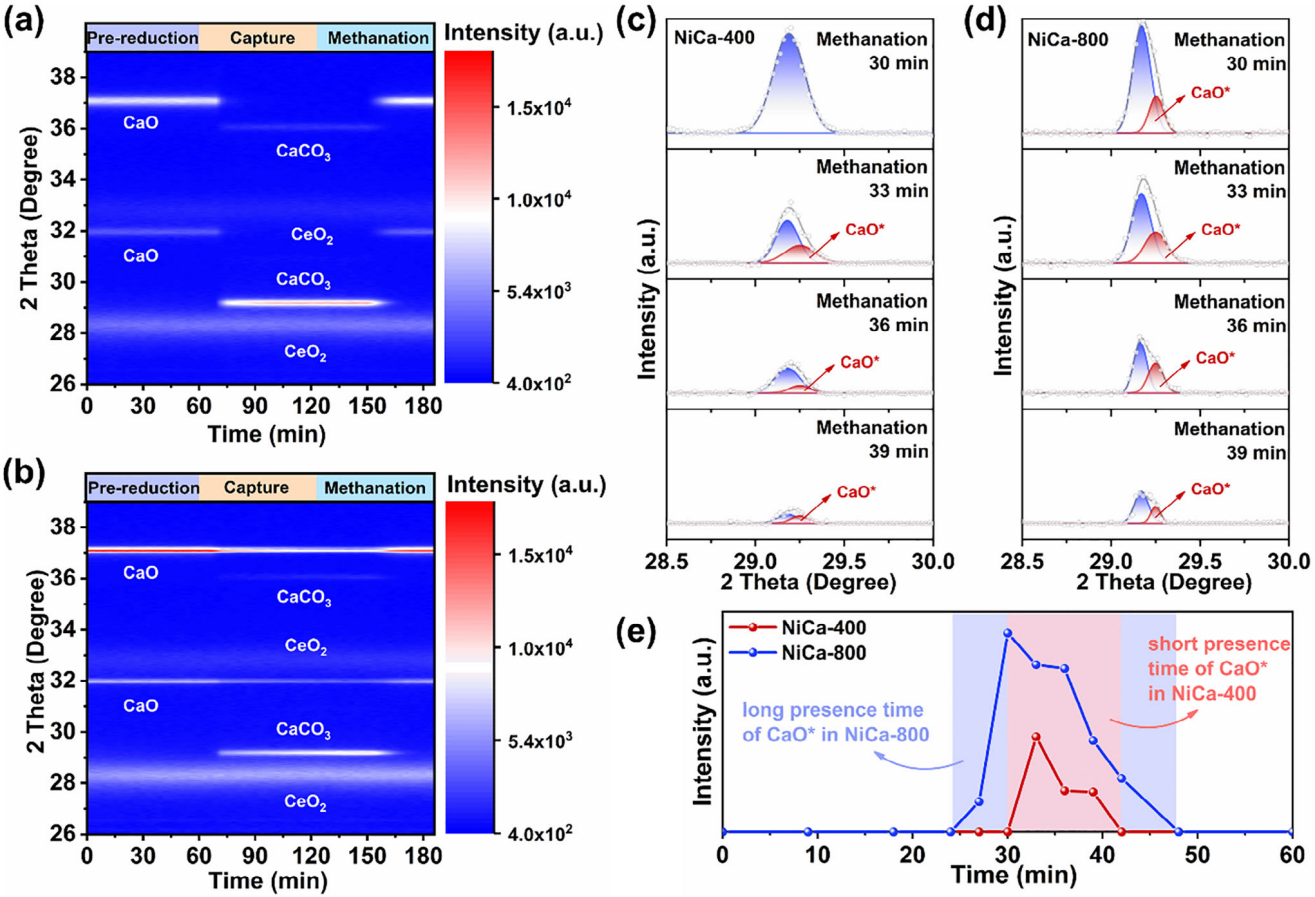
In situ XRD spectra of NiCa‐400 (a) and NiCa‐800 (b) in an ICCM cycle at 550 °C (pre‐reduction stage: 70% H_2_/N_2_, 60 min; capture stage: 20% CO_2_/N_2_, 60 min; methanation stage: 70% H_2_/N_2_, 60 min; 30 mL min^−1^). Results of peak fitting of crystalline phase transition in the methanation stage for NiCa‐400 (c) and NiCa‐800 (d). Variation of CaO* intensity during the methanation stage (e).

The evolution of the crystalline phases of CaCO_3_ and CaO was examined using in situ XRD at 550 °C, in an ICCM cycle. Since CaCO_3_ in NiCa‐400 can be converted entirely to CaO at ≈500 °C, only diffraction peaks of CaO are present in NiCa‐400 at the beginning of the pre‐reduction phase (**Figure** **4**a). Subsequently, carbonation of CaO occurs in the capture stage, resulting in CaCO_3_. Then, the regeneration of CaCO_3_ to CaO is achieved in the methanation stage. The crystal phase evolution of NiCa‐800 is similar to that of NiCa‐400 (Figure 4b). It is noteworthy that the diffraction peaks of CaO are always present in NiCa‐800 during the ICCM process, indicating that CaO is not completely carbonated, which is also consistent with the fact that NiCa‐800 has poor CO_2_ capture performance. The experimental peaks during the methanation process, where the CaCO_3_ to CaO conversion stage occurs, were analyzed for more discussion on CaO*. As shown in Figure 4c,d, the intensity of the prominent experimental peaks dominated by CaCO_3_ diminishes as the methanation reaction consumes CaCO_3_. The fitted peaks attributed to CaO* appeared and then gradually weakened. Figure 4e demonstrates the intensity variation of CaO* throughout the methanation stage, and it can be found that CaO* is only present in NiCa‐400 for a relatively short time. This indicates that the CaO synthesized via the low‐temperature hydrogen spillover strategy exhibits a kind of “genetic effect”, meaning that in subsequent reactions, CaCO_3_ can be converted into CaO more rapidly. We speculate that this may be related to the smaller size of the initially obtained CaO grains, which allows the subsequent nucleation growth of the grains to occur on a smaller scale and thus facilitates the rapid evolution of CaCO_3_ to CaO.^[^ ^37^ ^]^

### 2.5 Reaction Mechanism of DFMs

**Figure 5:**
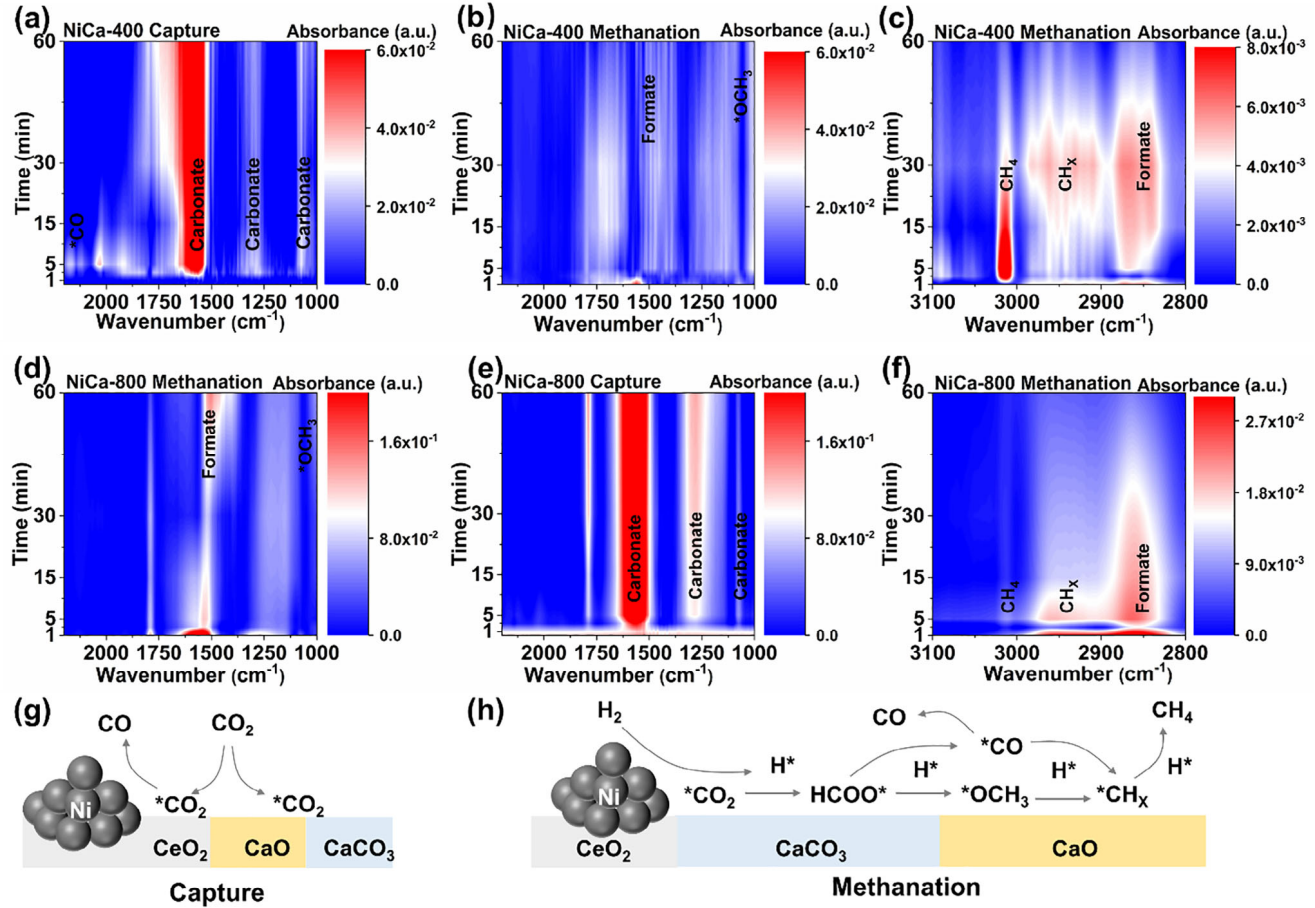
Time‐resolved 2D in situ DRIFTs patterns for NiCa‐400 (a–c) and NiCa‐800 (d–f) capture and methanation stages at 550 °C (capture stage: 20% CO_2_/N_2_, 60 min; methanation stage: 70% H_2_/N_2_, 60 min; 30 mL min^−1^). Mechanistic diagrams of CO_2_ capture (g) and methanation conversion (h).

To deepen the understanding of CO_2_ capture in situ methanation intermediates at the molecular level, we followed the changes of intermediates and products in an ICCM cycle by in‐situ diffuse reflectance infrared Fourier transform spectroscopy (in situ DRIFTs) (**Figure** **5**). For NiCa‐400, distinct carbonate bands appeared from 1574 to 1084 cm^−1^ during the CO_2_ capture phase, indicating that CaO adsorbed CO_2_ and generated carbonate species.^[^ ^38^ ^]^ The intensity of the carbonate absorption peaks gradually accumulated with the increase in capture time. At the same time, signals attributed to CO appeared at 2114 and 2176 cm^−1^, indicating partial CO_2_ cleavage at the oxygen vacancies of CeO_2_,^[^ ^15^ ^]^ but this phenomenon gradually diminished as the capture progressed. During the methanation stage, a gradual decay of the carbonate species was observed, implying that the H* produced by Ni/CeO_2_ interacts with CaCO_3_ and is converted to other intermediates. Typical formate groups were observed at 1520 and 2850 cm,^−1[^ ^39^ ^]^ and methoxy species at 1060 cm.^−1[^ ^17^ ^]^ The depletion of methoxy species was accompanied by the production of CH_4_ (3014 cm^−1^),^[^ ^40^ ^]^ further suggesting that methoxy is a crucial intermediate in methanation species. Meanwhile, an absorption peak caused by CH_X_ stretching vibration appeared between 2900 and 2990 cm,^−1[^ ^41^ ^]^ indicating that they are also transition state products of methanation. In addition, the weak signals near 2120 and 2170 cm^−1^ are attributed to *CO and gaseous CO, respectively.^[^ ^41^, ^42^ ^]^ Since CaCO_3_ is challenging to decompose at 550 °C, CO* comes from the decomposition of the formate, partially sustained hydrogenation produces CH_4_ desorption,^[^ ^11^, ^41^ ^]^ while less CO is directly desorbed as a by‐product. For NiCa‐800, the reaction mechanism is similar to that of NiCa‐400. Notably, during the methanation stage, the rates of consumption of carbonate species and CH_4_ production were significantly lower in NiCa‐800 than in NiCa‐400 (Figure S14, Supporting Information), further confirming the slower rates of calcium carbonate conversion and methanation in NiCa‐800. Based on our experiments, the reaction mechanisms of the CO_2_ capture stage and the methanation stage are summarized in Figure 5g,h.

### 2.6 Scale‐Up Experiments

**Figure 6:**
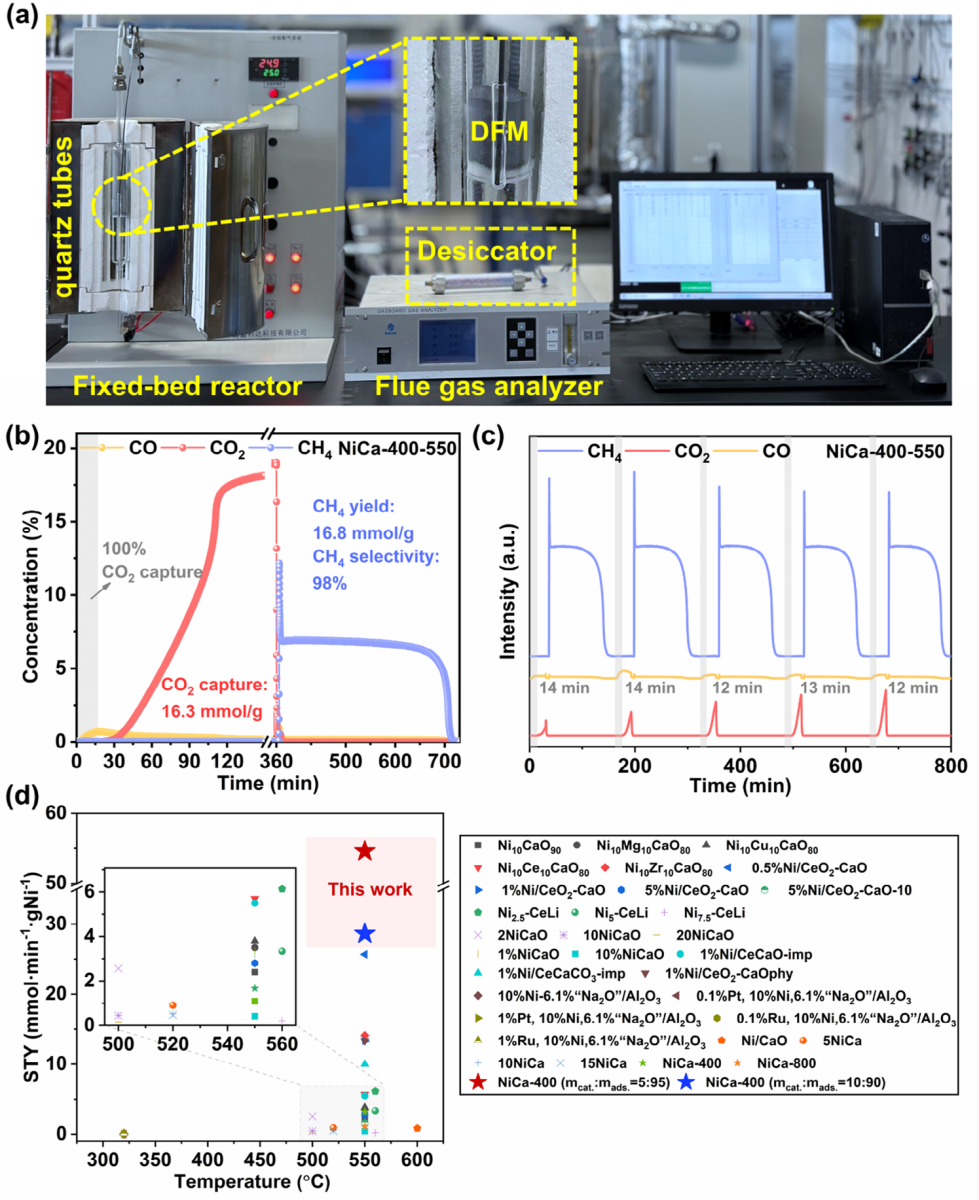
Scale‐up reactor unit (a); gas concentration changes curve at 550 °C (b) (capture stage: 20% CO_2_/N_2_, 360 min; methanation stage: 100% H_2_/N_2_, 360 min; 100 mL min^−1^); gas concentration changes for 5 ICCM breakthrough cycles test at 550 °C (c) (capture stage: 20% CO_2_/N_2_, 30 min; methanation stage: 100% H_2_/N_2_, 120 min; 100 mL min^−1^); comparison of STY values for DFMs with different catalyst and adsorbent ratios with other work (d).

We performed a scaled‐up performance evaluation to explore the feasibility of NiCa‐400R for large‐scale industrial applications. The test equipment used was consistent with the small pilot experiment, replacing a larger diameter reaction quartz tube (**Figure** **6**a). The first cycle had a CO_2_ capture of 16.3 mmol g^−1^, a CH_4_ yield of 16.8 mmol g^−1^, and a CH_4_ selectivity of 98% (Figure 6b). Compared with the first cycle of the small pilot experiment, the CO_2_ capture only decreased by 8.4%, and the CH_4_ yield only decreased by 2.3%.

CO_2_ breakthroughs are usually undesired in practical industrial applications because they cause new pollution. Therefore, we investigated the CO_2_ tolerance of NiCa‐400 (Figure 6c). CO_2_ can be guaranteed not to break through for 14 min in the first cycle (<5 s in the small pilot experiment). The CO_2_ concentration did not exceed 1% for the first 30 min of the capture stage. The CO_2_ captured was converted entirely to CH_4_ within 2 h, and the highest concentration of CH_4_ produced was 11%, which was close to twice the concentration produced by the small pilot experiment. After five breakthrough cycle tests, the CO_2_ breakthrough time was only 2 min earlier, and the CO_2_ capture exceeded 85% in the first 30 min.

The excellent performance of the scale‐up experiments demonstrates the possibility of further combining our proposed high‐activity DFM with industrial applications. However, the high cost of catalysts often becomes a limiting factor for industrial applications. To further reduce the cost, we tried to reduce the mixing ratio of catalyst and adsorbent while maintaining the performance. As the catalyst ratio decreases, the ICCM performance of DFMs is enhanced with increasing theoretical performance. Whereas, when the ratio of catalyst to adsorbent is <10:90, the selectivity and yield of CH_4_ start to decrease (Figure S15, Supporting Information). In addition, we compared the quantitative results of different catalyst and adsorbent ratios with other literature (Figure 6d; Table S3, Supporting Information). The STY values of DFMs for methanation near 550 °C are typically in the range of 0∼6 mmol/min/g_Ni_. By reducing the ratio of catalyst to DFMs, it is possible to increase the STY value up to 54.6 mmol/min/g_Ni_, which is a field‐leading level.

### 2.7 Techno‐Economic Evaluation

To further assess the scalability of the proposed strategy for industrial applications, a techno‐economic analysis was carried out using Aspen Plus (V11) software, while a CCU methanation model was constructed as a reference process (Figure S16, Supporting Information), with details of the process given in the Supporting Information.

Since the DFMs gradually deactivate as the process cycle progresses, the deactivation rates under two process conditions are defined in Tables S4 and S6 (Supporting Information). The results show that under the same operating conditions, the deactivated DFMs in the ICCU process are only 11% of those in the CCU process. In addition, since the ICCU process is an energy‐intensive process, it consumes only 21% of the energy used by CCU and is 12.9% more energy efficient than CCU (Table S7, Supporting Information). Meanwhile, since the ICCU process can be realized in a single reactor, the required equipment investment cost is only 41.9% of that of CCU (Table S8, Supporting Information), and the total annual investment is 23% lower than CCU (Table S9, Supporting Information).

**Figure 7:**
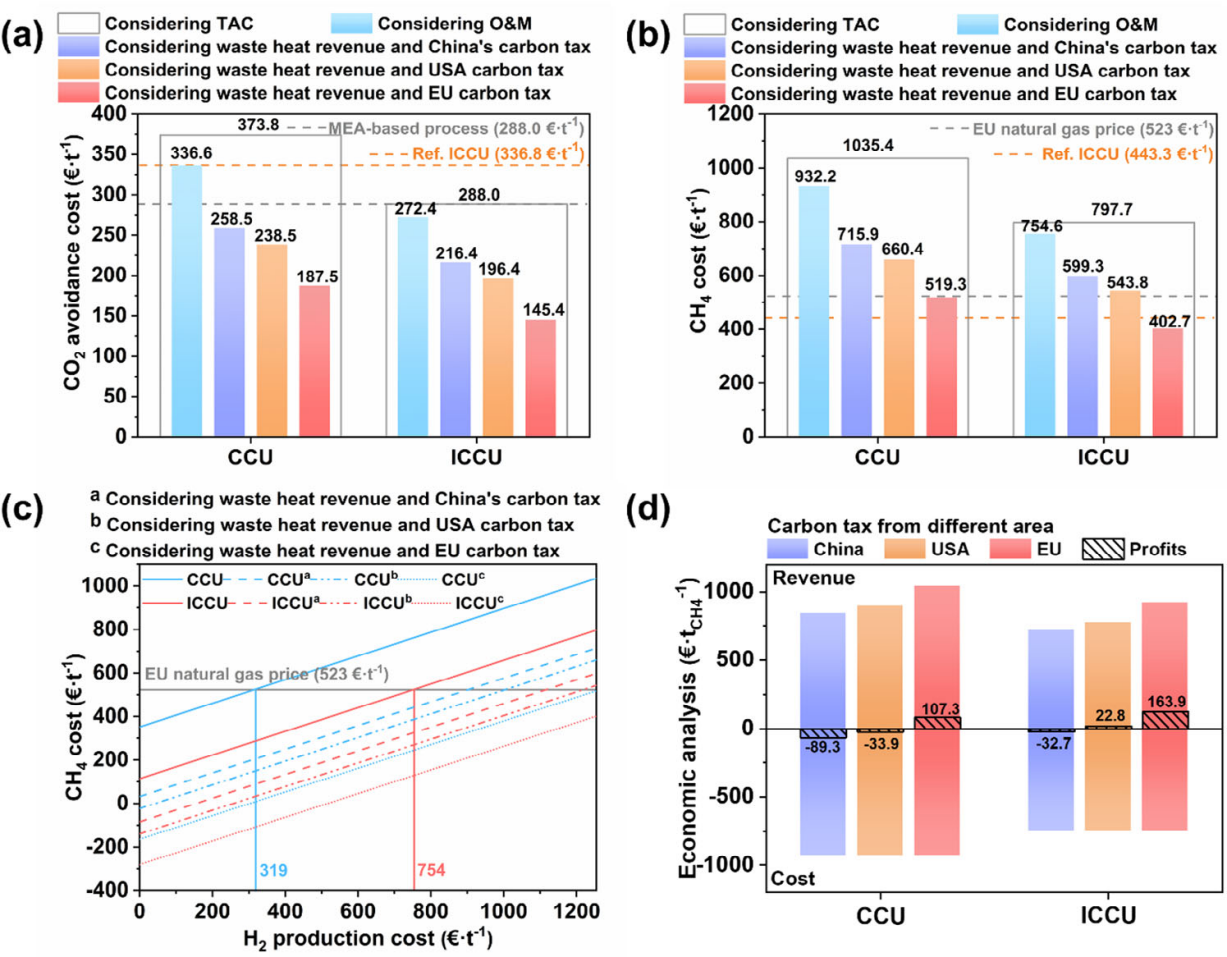
CO_2_ avoiding emission costs (a), CH_4_ costs (b), sensitivity analysis of H_2_ price to CH_4_ costs (c), profit and loss (d) for CCU and ICCU processes.

The CO_2_ avoidance cost of ICCU (288.0 €/t) was significantly lower than that of CCU (373.8 €/t). Further considering the power generation benefits of waste heat recovery and the EU carbon tax, the avoided cost of CO_2_ for ICCU is only 145.4 €/t, which is more competitive than both the MEA‐based CO_2_ capture process (288.0 €/t) and the reference ICCU process (336.8 €/t) (**Figure** **7**a).^[^ ^3^ ^]^ The cost per tonne of CH_4_ produced in the ICCU is 402.7 €, which is 22.5% lower than the CCU (519.3 €) and significantly lower than the EU natural gas price (523 €/t)^[^ ^43^ ^]^ and the reference process (443.3 €/t) (Figure 7b).^[^ ^3^ ^]^

The results of the sensitivity analysis of the impact of H_2_ prices on CH_4_ costs show that the ICCU will achieve equalization of CH_4_ production costs and market prices when the cost of hydrogen is reduced to 754 €/t, whereas for CCU it needs to be reduced to 319 €/t (Figure 7c). In addition, considering the higher strategic economic benefits of carbon abatement from the EU carbon tax, in the current context of high H_2_ costs, ICCU could gain 163.9 €/t CH_4_, which is 1.5 times more than CCU (Figure 7d). With the continuous development of sustainable development strategies and carbon‐neutral policies, we can forecast that the price of H_2_ will gradually decrease in the future, while the price of carbon tax will continue to rise. Therefore, our proposed strategy will be more attractive and promising for future industrial applications.

## 3 Conclusion

In summary, we propose the application of a low‐temperature hydrogen spillover decomposition strategy, which promotes the rapid evolution of CaCO_3_ into CaO, shortens the presence time of CaO* during the crystalline phase transition, and hinders the agglomeration of CaO particles. The smaller initial CaO particle size promotes mass transfer in the ICCM cycle and maintains the excellent long‐term performance of the DFMs with a “genetic effect”. The NiCa‐T with different calcination temperatures was preferred and matched with the optimal reaction temperature. At 550 °C, NiCa‐400 exhibited 17.8 mmol g^−1^ CO_2_ capture capacity and 17.2 mmol g^−1^ CH_4_ yield in the first ICCM cycle. It breaks through the decrease in the practical performance of CaO caused by the traditional synthesis method and achieves excellent performance with a near‐theoretical stoichiometric ratio. In addition, scale‐up experiments and techno‐economic analyses further clarify the feasibility of industrializing our proposed strategy, with a 79% reduction in energy consumption and a 23% saving in investment cost compared to CCU. This strategy provides a solution to improve the efficient resource utilization of carbonates in the food, construction, and cement industries, as well as a practical path to achieving carbon neutrality targets.

## 4 Experimental Section

### Material Preparation

Highly active calcium‐based adsorbents were synthesized by low‐temperature calcination based on conventional sol–gel. Typically, calcium nitrate tetrahydrate (Ca(NO_3_)_2_·4H_2_O), citric acid monohydrate (C_6_H_10_O_8_), and deionized water were mixed at a molar ratio of 1:1:40 and stirred in an oil bath at 90 °C until gelation. Then, it was dried at 130 °C overnight and calcined at T = 400∼800 °C for 4 h, named Ca‐T. The 10 wt.% Ni/CeO_2_ was synthesized by the impregnation method, and CeO_2_ nanorods were hydrothermally synthesized based on the scheme of Sun et al. The previous work describes the detailed steps.^[^ ^29^ ^]^ Ni/CeO_2_ and Ca‐T were physically mixed at a mass ratio of x:y, and the resultant DFMs were designated as NiCa‐T (m_cat._: m_ads._ = x:y). During this process, the mass of Ca‐T was calculated based on its CaO equivalent. Specifically, for z g Ca‐T (where T = 400, 500, or 600), according to containing 0.56z g CaO. To simplify the representation, when the mass ratio x:y was 1:1, the DFMs were directly denoted as NiCa‐T. Then, the DFMs were placed in a vertical fixed bed and pre‐reduced for 2 h at 550 °C in hydrogen. The reduced material was added with R after the name.

### Material Characterization

Dry gel foams were analyzed for weight loss on a thermogravimetric analyzer (HCT‐1, Beijing Hengjiu) to determine the material composition at different temperatures. The Flynn‐Wall‐Ozawa (FWO) method was used for the kinetic analysis, and a detailed introduction was provided in the previous work.^[^ ^29^ ^]^ The X‐ray diffraction (XRD) patterns were obtained using a Cu kα ray source and tested on a Rigaku Miniflex600 diffractometer. The material morphology was observed using a field emission scanning electron microscope (FESEM, Apreo S LoVac), and Pt sputtering was performed before observation. A field emission transmission electron microscope (FETEM, FEI Tecnai G2 F20 X‐Twin) equipped with an EDX was used to collect the imaging and elemental distribution of the material. The adsorption‐desorption isotherms were measured with a Micromeritics ASAP 2460 analyzer at 77 K. The materials were degassed at 150 °C for 4 h before the measurements. The specific surface area and pore size distribution of the samples were calculated according to the Brunauer–Emmet–Teller (BET) model and the Barrett–Joyner–Halenda (BJH) method, respectively. Detailed procedures for TPD, TPSR, in situ DRIFTs, in situ XRD, and ICCM testing are given in the Supporting Information.

## Conflict of Interest

The authors declare no conflict of interest.

## Author Contributions

L.W. performed the experiments, analyzed the data, and wrote the manuscript. R.H. conceived and designed the experiments and revised the manuscript. G.H. and H.Y. performed the in situ experimental tests; M.P. and Z.L. performed the economic analysis. C.S. and Q.L. supervised the experiments and critically revised the manuscript. All authors have read and approved the final manuscript.

## Supporting information



Supporting Information

## Data Availability

The data that support the findings of this study are available from the corresponding author upon reasonable request.

## References

[1] Z.‐K. Guo , S. Gao , S.‐X. Xiang , J.‐P. Wang , G.‐C. Mao , H.‐L. Jiang , B.‐X. Dong , Y.‐L. Teng , Chem. Eng. J. 2024, 481, 148599.

[2] M. S. Duyar , M. A. A. Treviño , R. J. Farrauto , Appl. Catal., B 2015, 168–169, 370.

[3] Z. Lv , H. Du , S. Xu , T. Deng , J. Ruan , C. Qin , Appl. Energy 2024, 355, 122242.

[4] C. Wang , H. Sun , X. Liu , X. Jin , Y. Feng , H. Shi , D. Wang , Y. Zhang , Y. Wang , Z. Yan , Fuel 2023, 345, 128238.

[5] P. Huang , Y. Guo , G. Wang , J. Yu , C. Zhao , X. Wang , T. Wang , Energy Fuels 2021, 35, 20185.

[6] Y. Yang , J. Liu , F. Liu , D. Wu , Fuel 2020, 276, 118093.

[7] H. Jiang , Q. Gao , S. Wang , Y. Chen , M. Zhang , J. CO2 Util. 2019, 31, 167.

[8] T. H. Nguyen , H. B. Kim , E. D. Park , Catalysts 2022, 12, 212.

[9] P. Unwiset , P. Kidkhunthod , Y. Poo‐arporn , K. C. Chanapattharapol , Appl. Catal., A 2022, 641, 118670.

[10] J.‐H. Woo , S. Jo , J.‐E. Kim , T.‐Y. Kim , H.‐D. Son , H.‐J. Ryu , B. Hwang , J.‐C. Kim , S.‐C. Lee , K. L. Gilliard‐AbdulAziz , Catalysts 2023, 13.

[11] Z. Lv , J. Ruan , W. Tu , X. Hu , D. He , X. Huang , C. Qin , Sep. Purif. Technol. 2023, 309, 123044.

[12] H. Sun , Y. Zhang , S. Guan , J. Huang , C. Wu , J. CO2 Util. 2020, 38, 262.

[13] M. A. Arellano‐Treviño , Z. He , M. C. Libby , R. J. Farrauto , J. CO2 Util. 2019, 31, 143.

[14] a) F. Kosaka , Y. Liu , S.‐Y. Chen , T. Mochizuki , H. Takagi , A. Urakawa , K. Kuramoto , ACS Sustainable Chem. Eng. 2021, 9, 3452;

[15] H. Sun , Y. Zhang , C. Wang , M. A. Isaacs , A. I. Osman , Y. Wang , D. Rooney , Y. Wang , Z. Yan , C. M. A. Parlett , F. Wang , C. Wu , Chem. Eng. J. 2022, 437, 135394.

[16] M. S. Duyar , S. Wang , M. A. Arellano‐Treviño , R. J. Farrauto , J. CO2 Util. 2016, 15, 65.

[17] X. Wu , R. Chang , M. Tan , L. Tao , Q. Fan , X. Hu , H. L. Tan , M. Åhlén , O. Cheung , W. Liu , Appl. Catal., B 2023, 338, 123053.

[18] A. Bermejo‐López , B. Pereda‐Ayo , J. A. González‐Marcos , J. R. González‐Velasco , J. CO2 Util. 2019, 34, 576.

[19] H. Sun , J. Wang , X. Liu , B. Shen , C. M. A. Parlett , G. Adwek , E. J. Anthony , P. T. Williams , C. Wu , J. Mater. Chem. A 2019, 7, 9977.

[20] a) E. T. Santos , C. Alfonsín , A. J. S. Chambel , A. C. Fernandes , A. P. S. Dias , C. I. C. Pinheiro , M. F. Ribeiro , Fuel 2012, 94, 624;

[21] a) S. Medina‐Carrasco , J. M. Valverde , Chem. Eng. J. 2022, 429, 132244;

[22] a) H. Liu , F. Pan , S. Wu , RSC Adv. 2019, 9, 26949; 35528570 10.1039/c9ra03611c PMC9070547

[23] E. P. Hyatt , I. B. Cutler , M. E. Wadsworth , J. Am. Ceram. Soc. 1958, 41, 70.

[24] D. Beruto , A. W. Searcy , J. Chem. Soc., Faraday Trans. 1 1974, 70, 2145.

[25] C. Rodriguez‐Navarro , E. Ruiz‐Agudo , A. Luque , A. B. Rodriguez‐Navarro , M. Ortega‐Huertas , Am. Minerlog. 2009, 94, 578.

[26] J. M. Valverde , S. Medina , Phys. Chem. Chem. Phys. 2015, 17, 21912. 26235797 10.1039/c5cp02715b

[27] D. Beruto , A. W. Searcy , Nature 1976, 263, 221.

[28] B. Sarrión , A. Perejón , P. E. Sánchez‐Jiménez , N. Amghar , R. Chacartegui , J. Manuel Valverde , L. A. Pérez‐Maqueda , Chem. Eng. J. 2021, 417, 127922.

[29] L. Wei , R. Han , M. Peng , Z. Li , C. Zhang , Q. Liu , Chem. Eng. J. 2024, 493, 152560.

[30] N. Czuma , K. Zarębska , M. Motak , M. E. Gálvez , P. Da Costa , Fuel 2020, 267, 117139.

[31] L. Chen , D. Liu , G. Wei , Energy Convers. Manage. 2024, 299, 117811.

[32] K. Ren , F. Jia , C. Zhang , E. Xing , Y. Li , Fuel 2023, 335, 127047.

[33] Y. Guo , S. Mei , K. Yuan , D. J. Wang , H. Liu , C. Yan , Y. W. Zhang , ACS Catal. 2018, 8, 6203.

[34] S. Xia , Z. Yuan , L. Wang , P. Chen , Z. Hou , Appl. Catalys. A‐general 2011, 403, 173.

[35] A. A. Scaltsoyiannes , A. A. Lemonidou , Chem. Eng. Sci. 2021, 243, 116797.

[36] a) J. Shen , X. Cheng , W. Wei , X. Tian , M. Ding , ACS Catal. 2025, 15, 2402;

[37] S. Ray , T. K. Bhattacharya , V. K. Singh , D. Deb , S. Ghosh , S. Das , Ceram. Int. 2021, 47, 858.

[38] L.‐P. Merkouri , J. L. Martín‐Espejo , L. F. Bobadilla , J. A. Odriozola , A. Penkova , T. Ramirez Reina , M. S. Duyar , J. Mater. Chem. A 2023, 11, 13209. 10.3390/nano13030506 PMC9921773 36770467

[39] Y. Yu , Y. M. Chan , Z. Bian , F. Song , J. Wang , Q. Zhong , S. Kawi , Int. J. Hydrogen Energy 2018, 43, 15191.

[40] Z. Zhang , C. Shen , K. Sun , X. Jia , J. Ye , C.‐j. Liu , J. Mater. Chem. A 2022, 10, 5792.

[41] a) L. Xu , Y. Cui , M. Chen , X. Wen , C. Lv , X. Wu , C.‐e. Wu , Z. Miao , X. Hu , Ind. Eng. Chem. Res. 2021, 60, 8056;

[42] S. Jo , J. H. Woo , T. Nguyen , J. E. Kim , T. Y. Kim , H.‐J. Ryu , B. Hwang , J. C. Kim , S.‐C. Lee , K. L. Gilliard‐Abdul‐Aziz , Energy Fuels 2023, 37, 19680.

[43] https://tradingeconomics.com/commodity/eu‐natural‐gas.

