# Supplementary material for: Modulating the Evolution of Metastable CaO* for the Near‐Theoretical Performance Breakthrough of Ni/CeO2‐CaO in Integrated CO2 Capture and Methanation
Source: Adv Sci (Weinh). 2025 Mar 26;12(26):2503086. doi: 10.1002/advs.202503086 (PMC12244995; doi:10.1002/advs.202503086)
Supplement: Supplementary file 1 — Supporting Information [file ADVS-12-2503086-s001.docx]

**Supporting Information**

**Modulation of the rapid evolution of metastable CaO* for the near-theoretical breakthrough of Ni/CeO_2_-CaO in integrated CO_2_ capture and methanation.**

Lifei Wei ^a, b^, Rui Han ^a, b*^, Gaoqi Han ^a, b^, Han Yan ^a, b^, Mingke Peng ^a, b^, Zhiyong Li ^a, b^, Chunfeng Song ^a, b^, Qingling Liu ^a, b^

*^a^ Tianjin Key Lab of Indoor Air Environmental Quality Control, School of Environmental Science and Technology, Tianjin University, Tianjin, 300350, China*

*^b^ State Key Laboratory of Engines, School of mechanical engineering, Tianjin University, Tianjin, 300350, China*

**Table of Contents**

[**1. Experimental Procedures 1**](#_Toc184071444)

[**2. Supporting figures and Tables 4**](#_Toc184071445)

[**3. Process simulation and economic evaluation 25**](#_Toc184071446)

[**4. Reference 37**](#_Toc184071447)

## 1. Experimental Section

CO_2_-programmed warming desorption (CO_2_-TPD) and hydrogen-programmed warming desorption (H_2_-TPD) were performed on an automated chemical adsorption flow analyzer (BSD-Chem-C200). Temperature-programmed surface reaction (TPSR) was performed on a vertical fixed bed. For CO_2_-TPD, 50 mg of reduced DFMs were taken and pretreated at 550 °C in 10% H_2_/Ar for 1 h at a total flow rate of 30 mL min^-1^. Subsequently, the samples were cooled to 50 °C and exposed to 20% CO_2_/N_2_ for 1 h. Then, the gas was switched to He for 1 h to remove physically adsorbed CO_2_. Finally, the sample was heated to 900 °C at a heating rate of 10 °C min^-1^. For H_2_-TPD, 50 mg of reduced catalyst and equal CaO equivalents of Ca-400 were taken, and another 50 mg of reduced catalyst was taken as a comparison sample. For differentiation, the mixture of the reduced catalyst and Ca - 400 is denoted as Ni/CeO_2_R - Ca400. The samples were pretreated at 250 °C, 10% H_2_/Ar for 1 h at a total flow rate of 30 mL min^-1^. Subsequently, the samples were cooled to 50 °C and exposed to 10% H_2_/Ar for 1 h. Then, the gas was switched to Ar for 1 h. Finally, the samples were heated to 600 °C at a ramp rate of 10°C min^-1^. CO_2_- TPSR and H_2_- TPSR were used to investigate the performance of the CO_2_ capture and methanation processes of the materials, respectively. For CO_2_- TPSR, 0.3 g of DFM was first reduced in a hydrogen atmosphere at 550 °C for 2 h. After cooling to room temperature, the temperature was raised to 1000 °C by passing 20% CO_2_/N_2_. For H_2_-TPSR, NiCa-400 was heated from room temperature to 1000 °C in a pure hydrogen atmosphere. NiCa-800 needed to be pre-reduced and fully carbonated (550 °C, 100% CO_2_,1h) before cooling to room temperature.

The reaction mechanism was deduced by in situ DRIFTs experiments on a Thermo Scientific Nicolet iS20. The materials were pre-reduced at 550 °C in 70% H_2_/N_2_ for 1 h. After N_2_ purge for 5 min, the materials were carbonated with 20% CO_2_/N_2_ for 1 h and then purged with N_2_ for 5 min. The feed gas was switched to 70% H_2_/N_2_ for methanation.

The crystalline phase evolution during the material reaction was obtained by in situ XRD on a Rigaku smartlab. XRD scans were recorded continuously at 26° ~ 2θ ~ 39 (10°/min). The temperature was raised to 550 °C in 70% H_2_/N_2_ and maintained for 1 h. Then, the gas path was switched to 20% CO_2_/N_2_ carbonation after a 5 min N_2_ purge for 1 h. After another 5 min N_2_ purge, the conversion was carried out in 70% H_2_/N_2_. For NiCa-400, in situ experiments were carried out in the N_2_ atmosphere, ramping up to 800 °C.

**ICCM test**

The performance of the materials was evaluated using a vertical fixed bed equipped with a flue gas analyzer. Approximately 0.3 g (0.15 g CaO equivalent adsorbent and 0.15 g catalyst) of DFMs were placed into a quartz tube. The temperature was raised to 550 °C under N_2_ with a 100 mL/min flow rate, and the flow rate was kept constant in subsequent stages. It was then pre-reduced in a hydrogen atmosphere for 2 h. Then N_2_ was passed to purge for 20 min while cooling/holding to 350, 450, and 550 °C. The feed gas was switched to 20% CO_2_/N_2_ for CO_2_ capture for 1 h. The feed gas was purged with N_2_ for 5 min, then switched to a pure hydrogen stream for methanation for 1 h. The above steps were repeated to investigate the cyclic stability performance of the materials. The calculation of CO_2_ capture capacity, CH_4_, CO and CO_2_ yield, CaCO_3_ conversion, CH_4_ selectivity and the average space-time yield (STY) of CH_4_ are described as follows:

$\text{CO}_{\text{2}}\text{ Capture Capacity }\left( \text{mmol }\text{g}_{\text{sorb}}^{\text{-1}} \right)\text{=}\frac{\int_{\text{0}}^{\text{t}_{\text{c}}} \left[ \text{F}_{\text{CO}_{\text{2}}}^{\text{0}}\text{-}\text{F}_{\text{CO}_{\text{2}}}^{\text{out}}\left( \text{t} \right) \right]\text{dt}}{\text{m}_{\text{s}}}$ (S1)

$\text{CH}_{\text{4}}\text{ Yield }\left( \text{mmol }\text{g}_{\text{catalyst}}^{\text{-1}} \right)\text{=(}\int_{\text{0}}^{\text{t}_{\text{m}}} \left[ \text{F}_{\text{CH}_{\text{4}}}^{\text{out}}\left( \text{t} \right) \right]\text{dt)/}\text{m}_{\text{c}}$ (S2)

$\text{CO Yield }\left( \text{mmol }\text{g}_{\text{catalyst}}^{\text{-1}} \right)\text{=(}\int_{\text{0}}^{\text{t}_{\text{m}}} \left[ \text{F}_{\text{CO}}^{\text{out}}\text{(t)} \right]\text{dt)/}\text{m}_{\text{c}}$ (S3)

$\text{CO}_{\text{2}}\text{ Yield }\left( \text{mmol }\text{g}_{\text{catalyst}}^{\text{-1}} \right)\text{=(}\int_{\text{0}}^{\text{t}_{\text{m}}} \left[ \text{F}_{\text{CO}_{\text{2}}}^{\text{out}}\text{(t)} \right]\text{dt)/}\text{m}_{\text{c}}$ (S4)

$\text{CaCO}_{\text{3}}\text{ Conversion}\text{=Carbon balance}\text{=}\frac{\text{CH}_{\text{4}}\text{ Yield+}\text{CO}_{\text{2}}\text{ Yield+CO Yield}}{\text{CO}_{\text{2}}\text{ capture capacity}}$ (S5)

$\text{CH}_{\text{4}}\text{ Selectivity=}\frac{\text{CH}_{\text{4}}\text{ Yield}}{\text{CH}_{\text{4}}\text{ Yield+CO Yield}}$ (S6)

$\text{STY=}{\int_{\text{0}}^{\text{t}_{\text{m}}} \left[ \left[ \text{F}_{\text{CH}_{\text{4}}}^{\text{out}}\left( \text{t} \right) \right]\text{dt} \right]}/{\text{M}_{\text{Ni}}\text{×}\text{t}_{\text{m}}}$ (S7)

Where $\text{F}_{\text{CO}_{\text{2}}}^{\text{0}}$ is the molar flow rate of exported CO_2_ (mmol/min) in the presence of no adsorbent. $\text{F}_{\text{CO}_{\text{2}}}^{\text{out}}\left( \text{t} \right)$, $\text{F}_{\text{CH}_{\text{4}}}^{\text{out}}\text{(t)}$, $\text{F}_{\text{CO}}^{\text{out}}\text{(t)}$ denote the molar flow rates of exported CO_2_, CH_4_ and CO (mmol/min), and M_Ni_ is the mass of Ni in 0.3 g DFMs, respectively. In addition, t_c_ and t_m_ (min) are the durations of CO_2_ capture and methanation conversion stages, m_s_ and m_c_ (g) are the masses of adsorbent and catalyst, respectively.

Scale-up experiments were carried out at the same flow rate as the small pilot experiment (100 mL/min), with 10 g of NiCa-400R capturing CO_2_ to saturation (6 h), and then H_2_ was fed until no more CH_4_ was produced (6 h). The yield calculations were similar to the small pilot experiments. The CO_2_ breakthrough limit of the material was also investigated in five cycles. In the first cycle, when the CO_2_ concentration breached 1% (30 min), the passage of CO_2_ was stopped immediately, and after N_2_ purging, H_2_ was fed until no more CH_4_ was produced (2 h). Subsequent cycles maintained the same capture and conversion durations as the first cycle.

## 2. Supporting figures and Tables





**Figure S1.** Thermal weight loss curve of the dried gel (21% O_2_/N_2_,100 mL/min).





**Figure S2.** XRD patterns of fresh adsorbents.


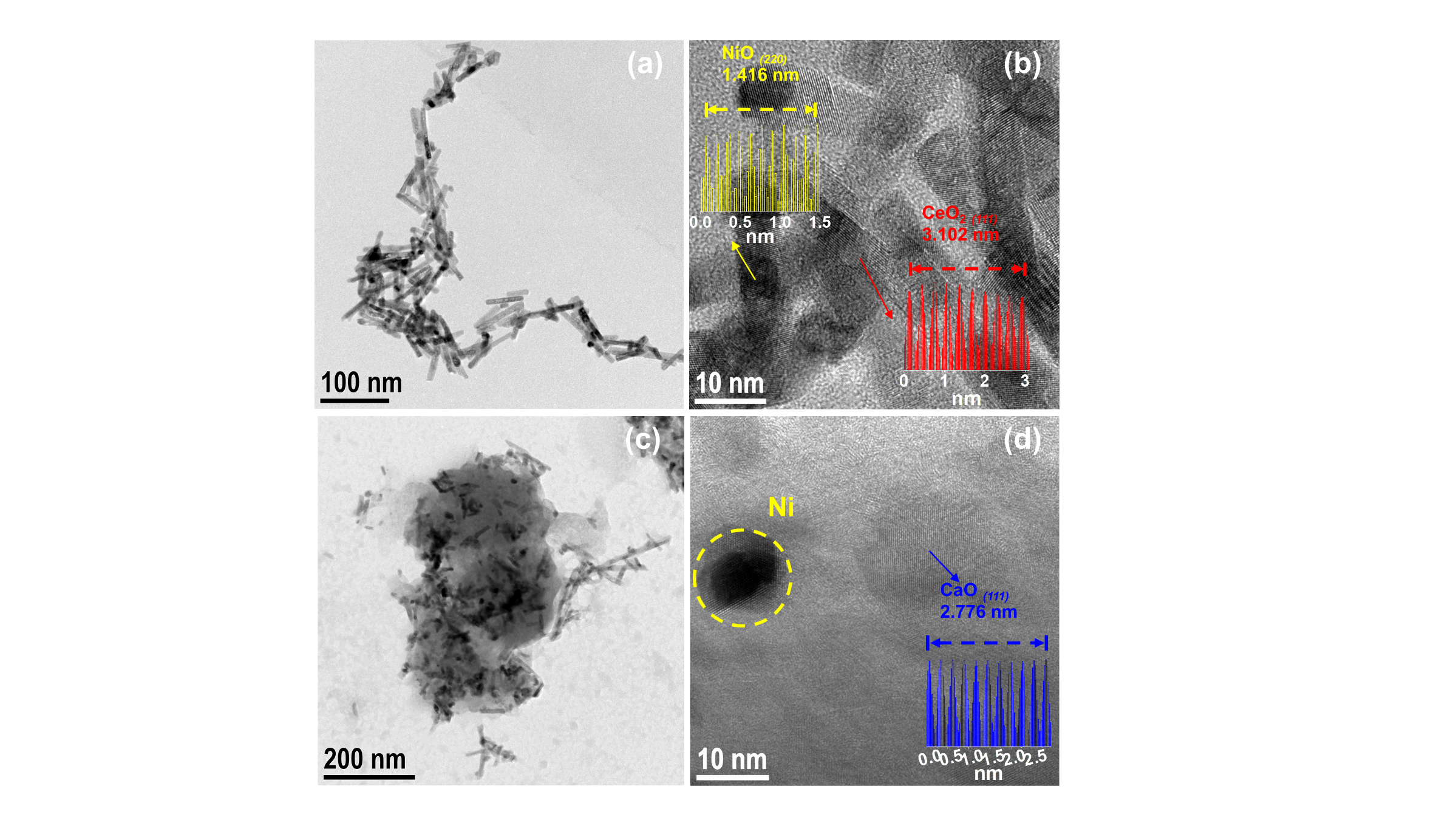


**Figure S3.** TEM and high-resolution TEM of Ni/CeO_2_R (a, b) and NiCa-400R (c, d).


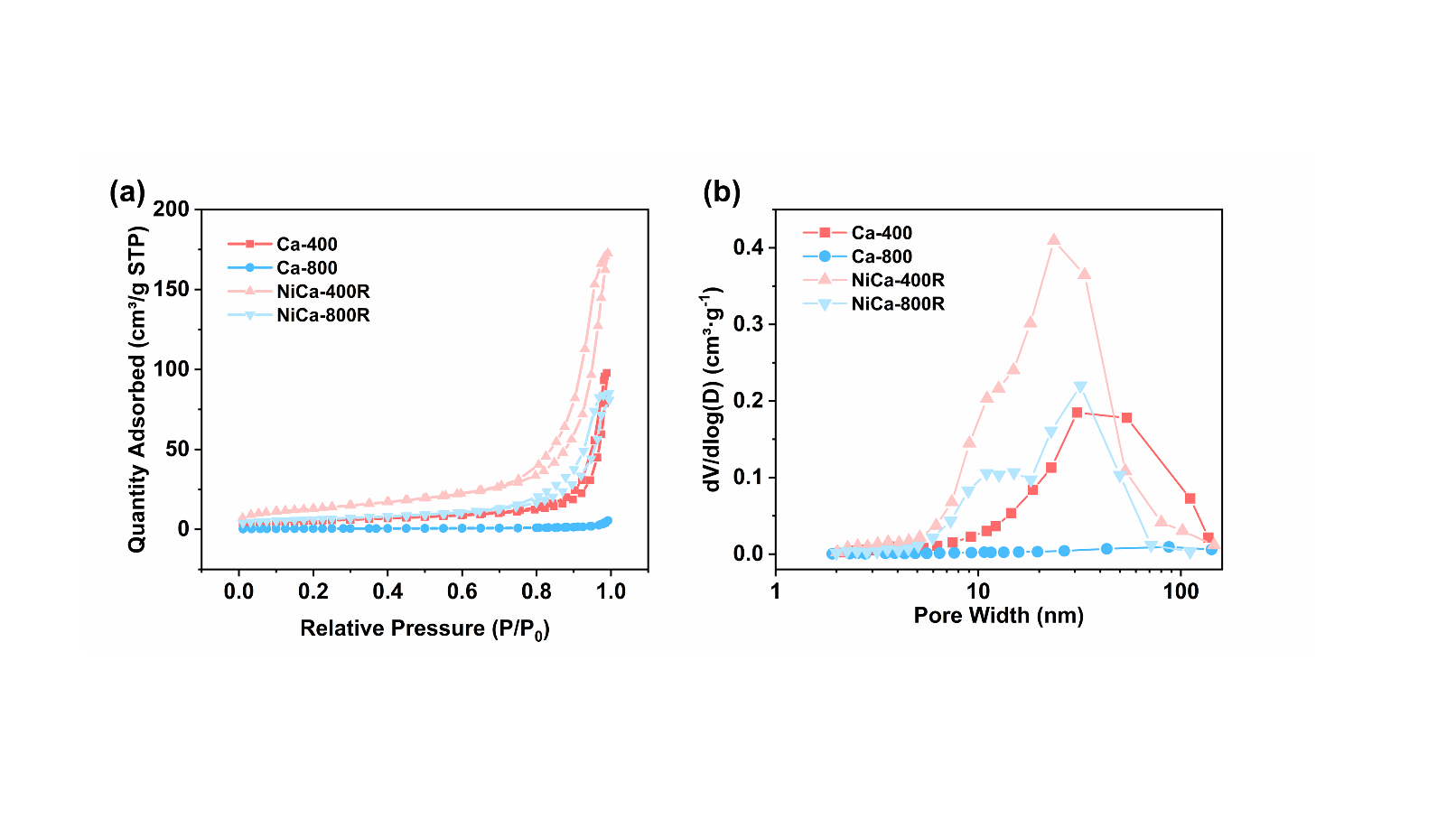


**Figure S4.** N_2_ adsorption-desorption isotherms (a) and pore size distribution calculated from the BJH desorption branch (b).


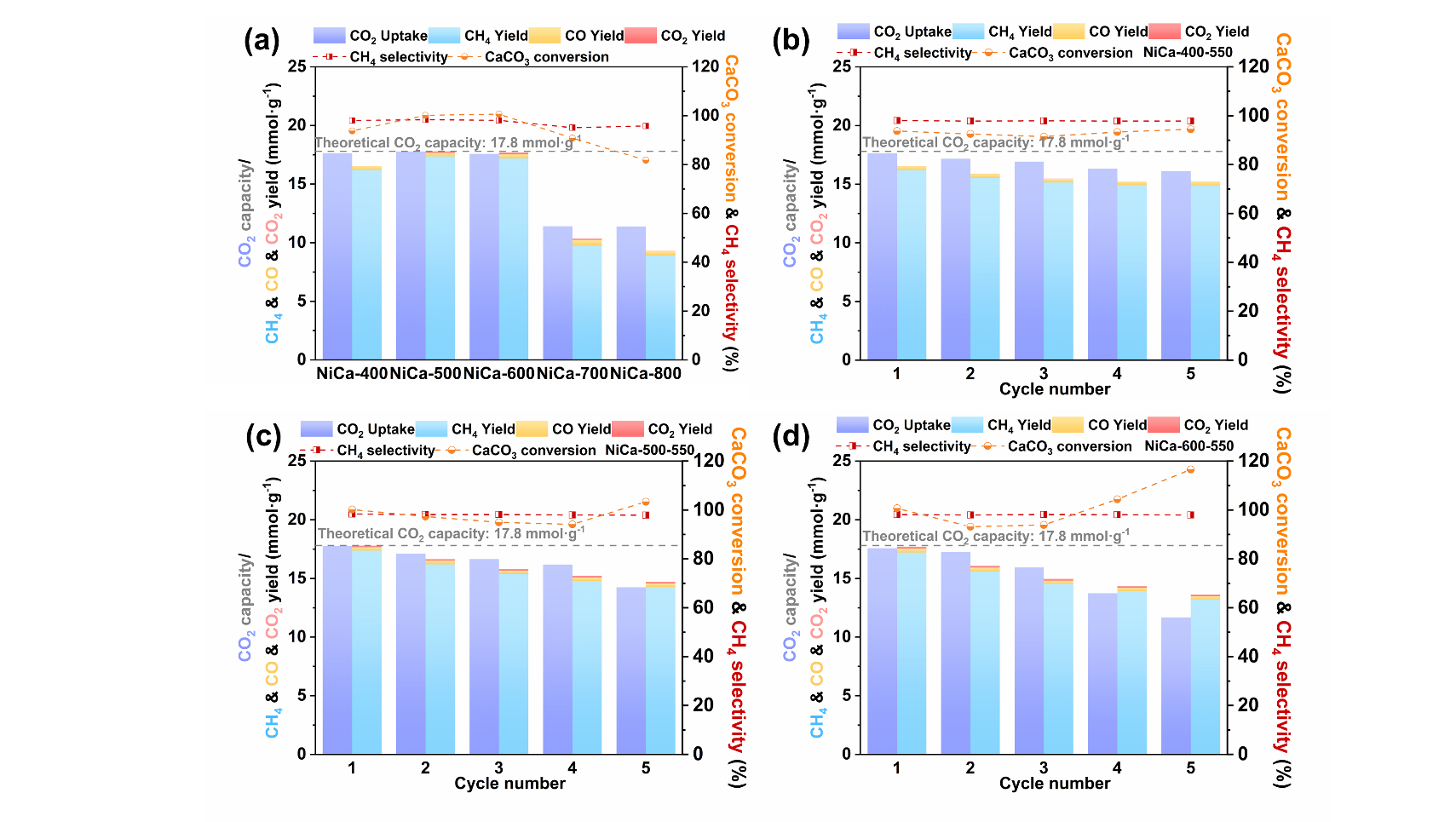


**Figure S5.** Comparison of the first cycle ICCM performance of NiCa-T at different calcination temperatures (a); 5-cycle ICCM performance of NiCa-400 (b), NiCa-500 (c), and NiCa-600 (d). (capture stage: 20% CO_2_/N_2_, 60 min; methanation stage: 100% H_2_, 60 min; 550 °C; 100 mL/min).


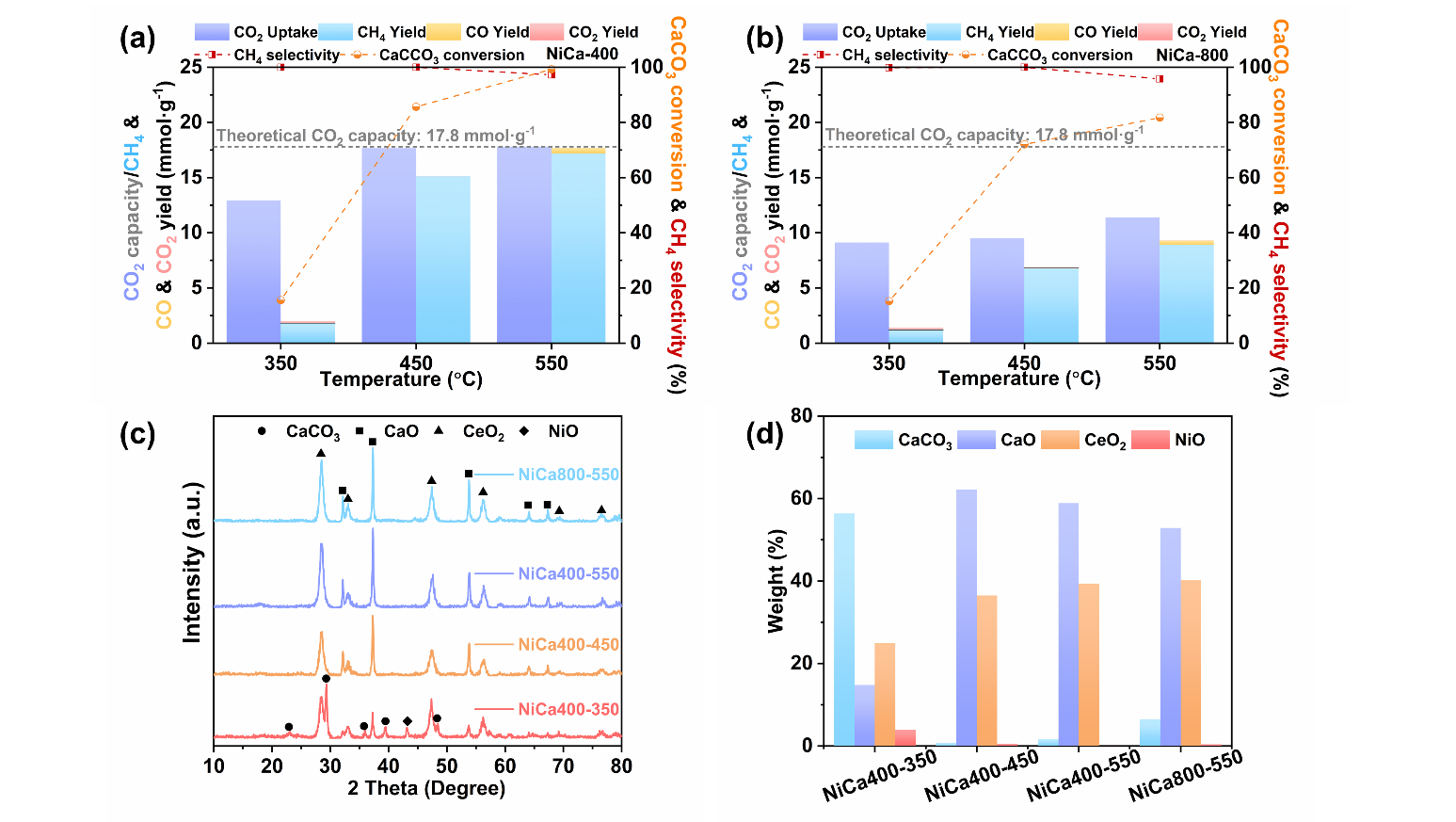


**Figure S6.** Comparison of the properties of NiCa-400 (a) and NiCa-800 (b) with different reaction temperatures (capture stage: 20% CO_2_/N_2_, 60 min; methanation stage: 100% H_2_, 60 min; 350 °C; 450 °C; 550 °C; 100 mL/min). XRD patterns (c) and composition (d) of DFM after 5 cycles at different reaction temperatures.





**Figure S7.** CO_2_-TPD of NiCa-400R and NiCa-800R.





**Figure S8.** H_2_-TPD of Ni/CeO_2_R and Ni/CeO_2_R-Ca400.


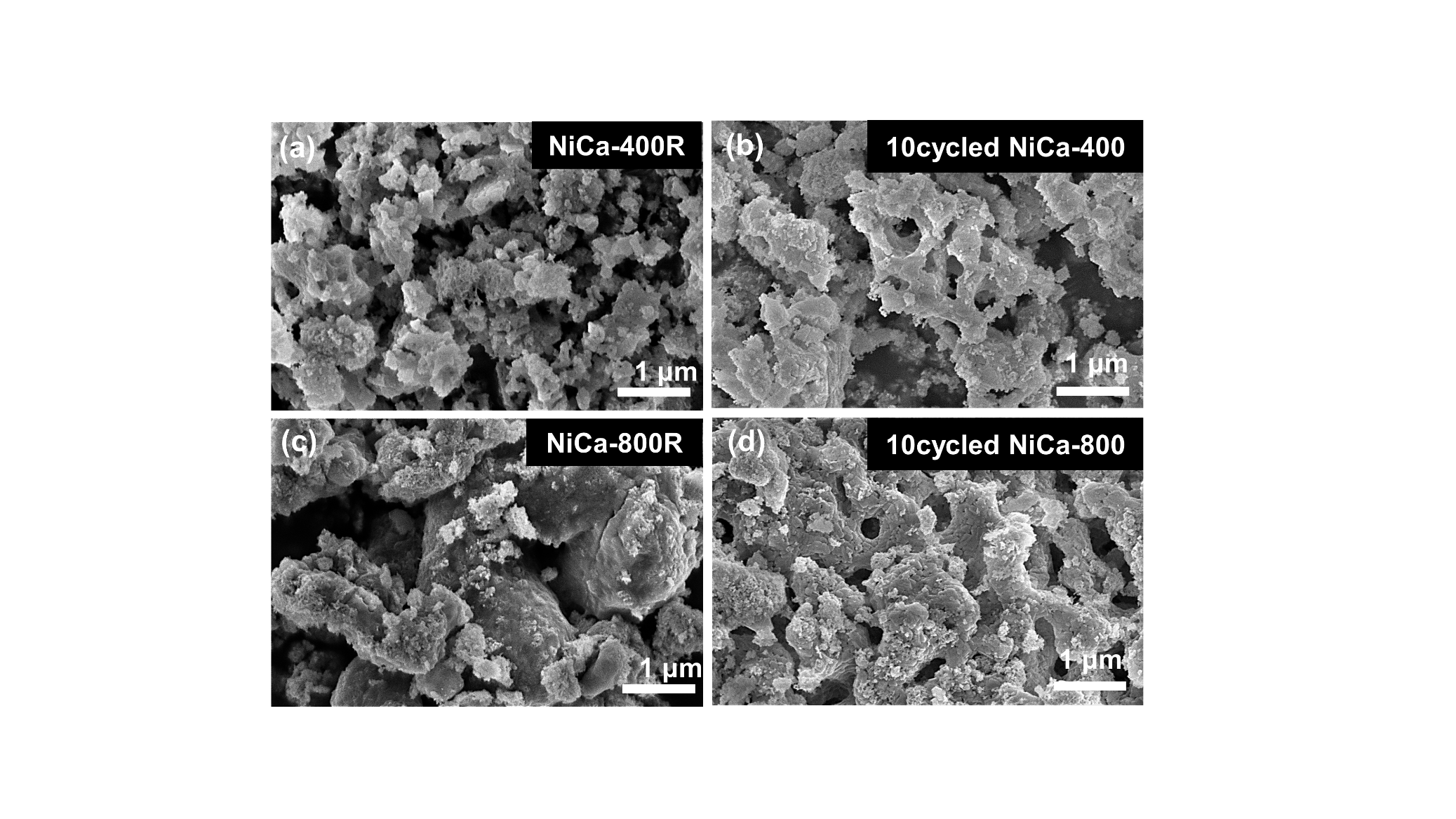


**Figure S9.** SEM images of NiCa-400R (a), NiCa-400after 10 ICCM cycles (b) and NiCa-800R (c), NiCa-800after 10 ICCM cycles (d).





**Figure S10.** XRD patterns of reduced and 10 cycled DFMs.


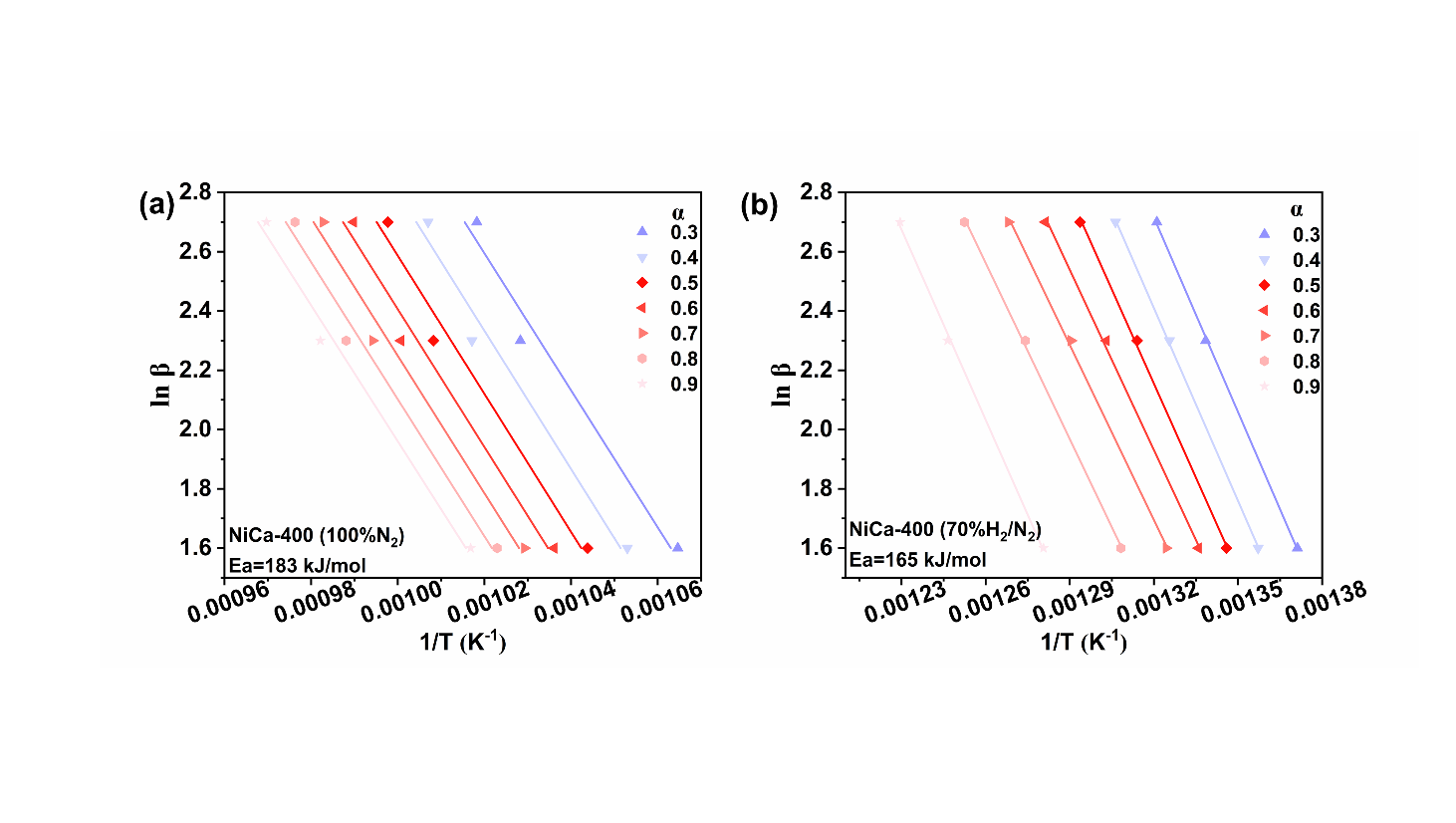


**Figure S11.** Kinetic analysis results of CaCO_3_ weight loss by NiCa-400 under N_2_ (a) and 70% H_2_/N_2_ (b) (β is the heating rate (°C/min), α is the conversion rate).





**Figure S12.** In situ XRD patterns of CaCO_3_ to CaO conversion stage in NiCa-400 in 70% H_2_/N_2_. (430 ~ 550 °C)





**Figure S13.** Thermal weight loss curve of Ca(OH)_2_.


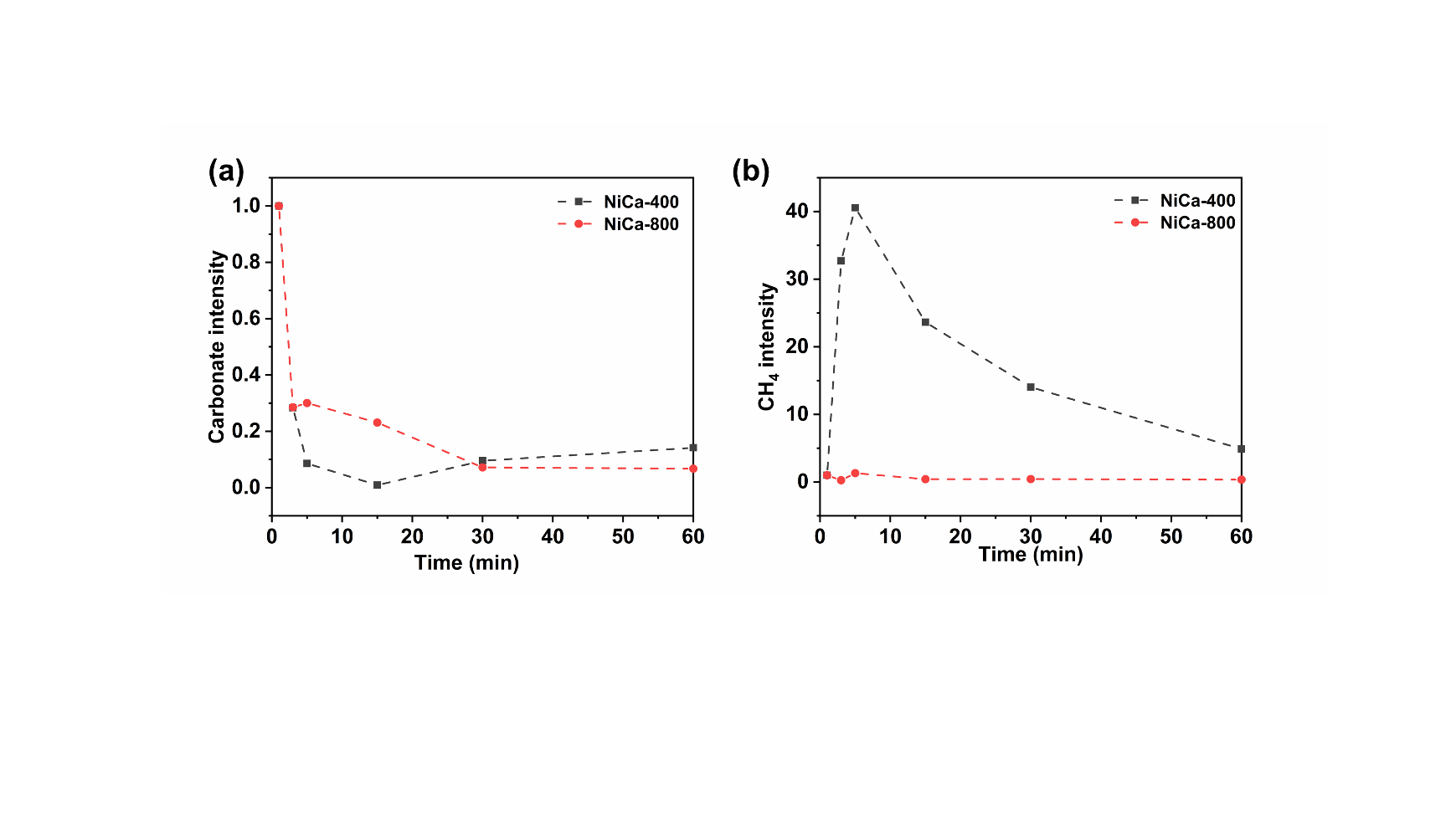


**Figure S14.** Changes in the intensity of carbonate and CH_4_ for NiCa-400 and NiCa-800 at the in-situ DRIFTS conversion stage.


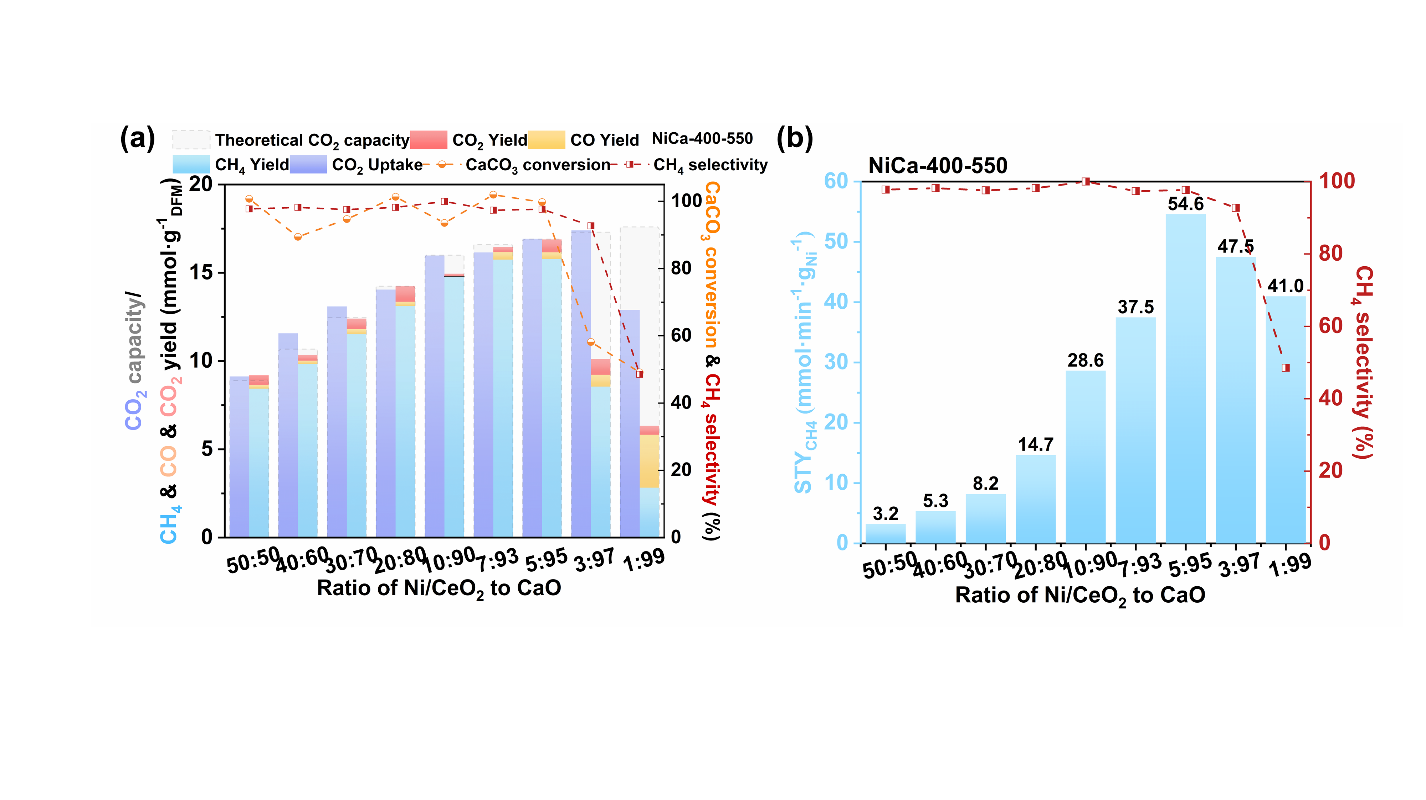


**Figure S15.** ICCM performance results (a) and STY quantification results (b) for DFMs with different catalyst and adsorbent ratios at 550 °C (capture stage: 20% CO_2_/N_2_, 60 min; methanation stage: 100% H_2_, 60 min; 550 °C; 100 mL/min).

**Table S1.** Structural properties of fresh adsorbent, reduced DFMs, and 10 cycled DFMs.

| **Sample** | **S_BET_ (m^2^/g)** | **V_p_ (cm^3^/g)** | **D_p_ (nm)** | **Grain size (nm)** | |
| --- | --- | --- | --- | --- | --- |
|  |  |  |  | **CaO** | **CeO_2_** |
| **Ca-400** | 19.0 | 0.15 | 27.0 | - | - |
| **Ca-800** | 1.74 | 0.01 | 24.9 | 81.6 | - |
| **Ni/CeO_2_** | 70.5 | 0.35 | 17.6 | - | - |
| **NiCa-400R** | 46.6 | 0.27 | 18.1 | 33.3 | 8.1 |
| **NiCa-800R** | 22.3 | 0.13 | 18.2 | 76.3 | 9.2 |
| **10 cycled NiCa-400** | 30.9 | 0.14 | 19.4 | 60.3 | 15.3 |
| **10 cycled NiCa-800** | 19.9 | 0.10 | 18.6 | 79.9 | 12.7 |

**Table S2.** Calculated activation energies of NiCa-400 for CaCO_3_ weight loss in N_2_ and 70% H_2_/N_2_ (FWO method)

| **α** | **NiCa-400** | |
| --- | --- | --- |
|  | **N_2_** | **70%H_2_** |
| **0.3** | 183 | 173 |
| **0.4** | 184 | 171 |
| **0.5** | 184 | 167 |
| **0.6** | 184 | 160 |
| **0.7** | 183 | 156 |
| **0.8** | 183 | 157 |
| **0.9** | 181 | 169 |
| $\bar{\text{Ea}}$ | 183 | 165 |

**Table S3.** Summary of STY values for DFMs with different catalyst and adsorbent ratios in comparison with other works.

| **Material** | **Temperature (℃)** | **Reaction condition** | **STY(mmol/min/g_Ni_)** | **Cycle** | **Ref.** |
| --- | --- | --- | --- | --- | --- |
| Ni_10_CaO_90_ | 550 | 15%CO_2_/N_2_, 30min;100%H_2_, 143min | 2.40 | 1 | ^[1]^ |
| Ni_10_Mg_10_CaO_80_ | 550 | 15%CO_2_/N_2_, 30min;100%H_2_, ~122min | 3.50 | 1 |  |
| Ni_10_Cu_10_CaO_80_ | 550 | 15%CO_2_/N_2_, 30min;100%H_2_, ~108min | 3.80 | 1 |  |
| Ni_10_Ce_10_CaO_80_ | 550 | 15%CO_2_/N_2_, 30min;100%H_2_, ~86min | 5.70 | 1 |  |
| Ni_10_Zr_10_CaO_80_ | 550 | 15%CO_2_/N_2_, 30min;100%H_2_, ~40min | 14.10 | 1 |  |
| 0.5%Ni/CeO_2_-CaO | 550 | 15%CO_2_/N_2_, 60min;100%H_2_, 60min | 25.67 | 1 | ^[2]^ |
| 1%Ni/CeO_2_-CaO | 550 | 15%CO_2_/N_2_, 60min;100%H_2_, 60min | 13.33 | 1 |  |
| 5%Ni/CeO_2_-CaO | 550 | 15%CO_2_/N_2_, 60min;100%H_2_, 60min | 2.80 | 1 |  |
| 5%Ni/CeO_2_-CaO-10 | 550 | 15%CO_2_/N_2_, 60min;100%H_2_, 60min | 1.67 | 10 |  |
| Ni_2.5_-CeLi | 560 | 15%CO_2_/N_2_, 30min;100%H_2_, 30min | 6.13 | 1 | ^[3]^ |
| Ni_5_-CeLi | 560 | 15%CO_2_/N_2_, 30min;100%H_2_, 30min | 3.33 | 1 |  |
| Ni_7.5_-CeLi | 560 | 15%CO_2_/N_2_, 30min;100%H_2_, 30min | 0.21 | 1 |  |
| 2NiCaO | 500 | 10%CO_2_,10%H_2_O, 120min; 90%H_2_, ~115min | 2.56 | 1 | ^[4]^ |
| 10NiCaO | 500 | 10%CO_2_,10%H_2_O, 120min; 90%H_2_, ~200min | 0.45 | 1 |  |
| 20NiCaO | 500 | 10%CO_2_,10%H_2_O, 120min; 90%H_2_, ~300min | 0.17 | 1 |  |
| 1%NiCaO | 550 | 15%CO_2_/N_2_, 60min;100%H_2_, 60min | 3.3 | 1 | ^[5]^ |
| 10%NiCaO | 550 | 15%CO_2_/N_2_, 60min;100%H_2_, 60min | 0.42 | 1 |  |
| 1%Ni/CeCaO-imp | 550 | 15%CO_2_/N_2_, 60min;100%H_2_, 60min | 5.50 | 1 |  |
| 1%Ni/CeCaCO_3_-imp | 550 | 15%CO_2_/N_2_, 60min;100%H_2_, 60min | 10.00 | 1 |  |
| 1%Ni/CeO_2_-CaOphy | 550 | 15%CO_2_/N_2_, 60min;100%H_2_, 60min | 13.30 | 1 |  |
| 10%Ni-6.1%“Na_2_O”/Al_2_O_3_ | 320 | 6.66%CO_2_/N_2_, 30min; 13.26%H_2_/N_2_, 360min | 0.0077 | 1 | ^[6]^ |
| 0.1%Pt, 10%Ni,6.1%“Na_2_O”/Al_2_O_3_ | 320 | 7.5%CO_2_, 4.5%O_2_, 15%H_2_O/N_2_, 20min; 15%H_2_/N_2_, 60min | 0.037 | 1 | ^[7]^ |
| 1%Pt, 10%Ni,6.1%“Na_2_O”/Al_2_O_3_ | 320 | 7.5%CO_2_, 4.5%O_2_, 15%H_2_O/N_2_, 20min; 15%H_2_/N_2_, 60min | 0.043 | 1 |  |
| 0.1%Ru, 10%Ni,6.1%“Na_2_O”/Al_2_O_3_ | 320 | 7.5%CO_2_, 4.5%O_2_, 15%H_2_O/N_2_, 20min; 15%H_2_/N_2_, 45min | 0.071 | 1 |  |
| 1%Ru, 10%Ni,6.1%“Na_2_O”/Al_2_O_3_ | 320 | 7.5%CO_2_, 4.5%O_2_, 15%H_2_O/N_2_, 20min; 15%H_2_/N_2_, 30min | 0.13 | 1 |  |
| Ni/CaO | 600 | 10 vol% CO_2_, 10 vol%H_2_O, N_2_ balance, 120min; 90%H_2_/N_2_, 180min | 0.83 | 1 | ^[8]^ |
| 5NiCa | 520 | 10%CO_2_/Ar, 1min; 10%H_2_/Ar, 2min | 0.91 | 1 | ^[9]^ |
| 10NiCa | 520 | 10%CO_2_/Ar, 1min; 10%H_2_/Ar, 2min | 0.525 | 1 |  |
| 15NiCa | 520 | 10%CO_2_/Ar, 1min; 10%H_2_/Ar, 2min | 0.473 | 1 |  |
| NiCa-400 (m_cat._ : m_ads。_=5:95) | 550 | 20%CO_2_/N_2_, 60min;100%H_2_, 60min | 54.59 | 1 | This work |
| NiCa-400 (m_cat._ : m_ads._=10:90) | 550 | 20%CO_2_/N_2_, 60min;100%H_2_, 60min | 28.65 | 1 | This work |
| NiCa-400 | 550 | 20%CO_2_/N_2_, 60min;100%H_2_, 60min | 3.2 | 1 | This work |
| NiCa-800 | 550 | 20%CO_2_/N_2_, 60min;100%H_2_, 60min | 1.09 | 1 | This work |

## 3. Process simulation and economic evaluation

The reference plant is a 1000MW coal-fired power plant with a thermal efficiency of 49.4% and a coal consumption of 248.86 kg/MW⋅h. After synergistic removal of pollutants, the flue gas has a temperature of 80 °C and a flow rate of 568.5 kg/s. Neglecting trace impurity fractions, the flue gas consists mainly of 20% CO_2_ and 80% N_2_, with CO_2_ acting as a source of CO_2_ for the CCU and the ICCU. The physical properties of the gas, adsorbent, and catalyst were calculated using the Peng-Robinson-Boston-Mathias (PR-BM) equation of state. The Aspen models of the CCU and ICCU are shown in **Figure S16**.

**(1) CCU and ICCU process assumptions and descriptions**

The CCU process consists of three main reactors simulating CO_2_ capture, desorption, and methanation processes, respectively. The flue gas first enters the CO_2_ capture unit (CR), where it is captured by CaO at 550 °C and then passes through a cyclone to achieve gas-solid separation with 100% separation efficiency. The clean flue gas is cooled down to 300 °C by a heat exchanger and discharged into the atmosphere.CaCO_3_ formed after carbonization of CaO desorbs CO_2_ in a calcination reactor (CALR) at 950 °C. The CO_2_ and regenerated CaO are separated by a cyclone separator. Considering that there is a certain degree of deactivation of CaO during the cycle, a splitter is set up to move the deactivated adsorbent out of the cycle. The remaining CaO is reused in the next cycle. The desorbed CO_2_ is cooled to 25 °C and then compressed and transported through a variable pressure unit. The pressure is then regulated to atmospheric pressure by a control valve and enters the methanation reactor (MR). At the same time, H_2_ was passed into the methanation reactor, and a slight excess of hydrogen was passed in order to ensure the complete reaction so that the molar ratio of H_2_ and CO_2_ was 4.1. CO_2_ was hydrogenated to methane at 550°C and then passed through a gas-liquid separation device to remove water vapor from the gas stream, and finally, the target product CH_4_ was obtained.

The ICCU process and simulation parameters are similar to the CCU. It is worth noting that ICCU can realized in practice with only one reactor. To demonstrate the process more visually, a virtual reactor and two virtual cyclone separators were added to the simulation. The two reactors simulate CO_2_ capture (CR) and in situ methanation (MR), respectively. In addition, since the ICCU is a capture-to-conversion integrated process, there is no need to set up the compression and transport processes. Detailed parameter settings are shown in **Table S4** and **S5.**

**(2) Economic evaluation assumptions and methods**

The total annual cost (TAC) consists of the total annual capital cost (ACC) and the annual operation and maintenance cost (O&M) (**Equation S8**) ^[10]^.

TAC = ACC + O&M (S8)

ACC is calculated from the total capital investment cost (C_total_) and the recovery factor (CRF) (**Equation S9**). The items included in C_total_ are shown in **Table S8 and S9.** CRF is obtained from **Equation S10** ^[11]^, where i is the rate of return, which is set to be 10 %. Where n is the plant lifetime, set to 25 years. The CH_4_ cost and the cost of CO_2_ avoidance (CAC) are obtained through **Equation S11** and **S12** ^[12]^.

ACC=CRF ×C_total_ (S9)

$\text{CRF=}\frac{\text{i(1+i)}^{\text{n}}}{\text{(1+i)}^{\text{n}}\text{-1}}$ (S10)

$\text{Cost of }\text{CH}_{\text{4}}\text{= }\frac{\text{TAC}}{\text{Annual }\text{CH}_{\text{4}}\text{ production}}$ (S11)

$\text{CAC= }\frac{\text{TAC}}{\text{Annual }\text{CO}_{\text{2}}\text{ emission reduction}}$ (S12)

A profit and loss analysis of the process is useful for assessing the revenue per tonne of CH_4_, which is a key factor to be considered for future industrialization. Costs mainly consider input material costs, utilities and other O&M costs, while revenues include product CH_4_, revenue from waste heat recovery and carbon tax, with associated costs and carbon tax prices shown in **Table S10.**


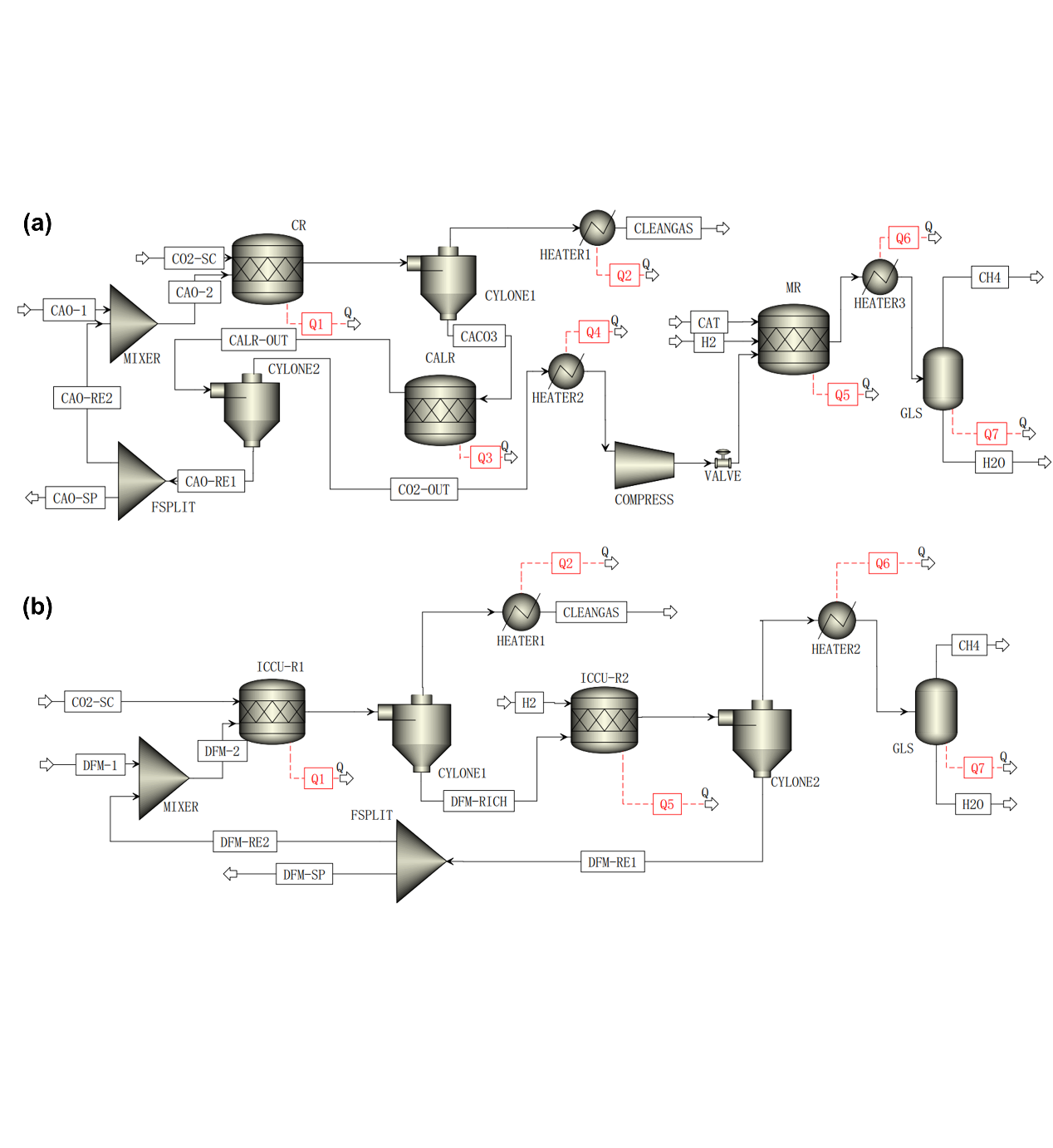


**Figure S16.** Process model of CCU (a) and ICCU (b) by Aspen Plus.

**Table S4.** CCU and ICCU process unit models and parameters.

| **Unit** | **Aspen model** | **Parameter** | **CCU** | **ICCU** |
| --- | --- | --- | --- | --- |
| CR/ICCU-R1 | Rstoic | Temperature (℃) | 550 | 550 |
|  |  | Pressure (bar) | 1.01 | 1.01 |
|  |  | CO_2_ capture efficiency (%) ^a^ | 95 | 95 |
| CYLONE | SSplit | Separation efficiency (%) | 100 | 100 |
| CALR | Rstoic | Temperature (℃) | 550 | - |
|  |  | Pressure (bar) | 1.01 | - |
|  |  | CaCO_3_ conversion (%) ^b^ | 100 | 94 |
| COMPRESS | Compr | Output pressure（bar） | 70 | - |
|  |  | Isentropic efficiency (%) | 100 | - |
| MR/ICCU-R2 | Rstoic | Temperature (℃) | 550 | 550 |
|  |  | Pressure (bar) | 1.01 | 1.01 |
|  |  | CO_2_ conversion (%) ^c^ | 99 | 99 |
| FSPLIT | Fsplit | Diversion rate (SP) (%) ^d^ | 5 | 0.50 |

^a)^ Scale-up results show that CO_2_ capture efficiency can reach 95% when the CaO to CO_2_ molar ratio is 3.3.

^b)^ For CCU, calcination at 950 °C resulted in 100% CaCO_3_ conversion. For ICCU, the conversion of CaCO_3_ was 94% when the mass ratio of adsorbent to catalyst was 9:1. To simulate the CO_2_ in-situ conversion process, CaCO_3_ decomposition was carried out as the first step reaction in ICCU-R_2_, and the actual reaction process was a direct gas-solid reaction between H_2_ and CaCO_3_.

^c)^ For CCU, the CO_2_ conversion was 99% when the CO_2_:H_2_ molar ratio was 1:4 ^[13]^. For ICCU, the CO_2_ conversion was 99% when the adsorbent to catalyst mass ratio was 9:1.

^d)^ The CCU process uses a fluidized bed reactor with an empirical adsorbent loss rate of 5%. For ICCU, the loss rate is set at 0.5% because the cycle can be completed in the same reactor only ^[14]^.

**Table S5.** CCU and ICCU process flow stream input parameters.

| **Stream** |  | **CCU** | **ICCU** |
| --- | --- | --- | --- |
| CO_2_-SC | Temperature (℃) | 80 | 80 |
|  | Pressure (bar) | 1.01 | 1.01 |
|  | Mass flow (kg/s) | 568.5 | 568.5 |
|  | CO_2_ content | 20 vol% | 20 vol% |
| CAO-2 | Temperature (℃) | 25 | - |
|  | Pressure (bar) | 1.01 | - |
|  | Mass flow (kg/s) | 668.56 | - |
| DFM-2 | Temperature (℃) | - | 25 |
|  | Pressure (bar) | - | 1.01 |
|  | Mass flow (kg/s) | - | 742.8 |
|  | m_cat_: m_ads_ | - | 1:9 |
| H_2_ | Temperature (℃) | 25 |  |
|  | Pressure (bar) | 1.01 | 1.01 |
|  | Mass flow (kg/s) | 29.7 | 29.7 |
| CAT | Temperature (℃) | 25 | - |
|  | Pressure (bar) | 1.01 | - |
|  | Mass flow (kg/s) | 74.3 | - |

**Table S6.** Summary of output results for CCU and ICCU.

| **Parameter** | **CCU** | **ICCU** |
| --- | --- | --- |
| CH_4_ produced (kg/s) | 54.6 | 54.6 |
| Clean flue gas (kg/s) | 409.3 | 409.3 |
| Deactivated absorbent (kg/s) | 33.4 | 3.3 |
| Deactivated catalyst (kg/s) ^a^ | 0.0579 | 0.367 |

^a)^ For CCU, the catalyst deactivation rate is based on 1.06 kg of deactivation per ton of product produced ^[15]^.

**Table S7.** Summary of energy balance for CCU and ICCU units.

| **Source of heat** |  | **CCU** | **ICCU** |
| --- | --- | --- | --- |
| CO_2_ capture reactor (Q1) | MW | -544.16 | - |
| ICCU Reactor 1 (Q1) | MW | - | -315.55 |
| Clean gas (Q2) | MW | -114.57 | -144.57 |
| Calcination Reactor (Q3) | MW | 933.93 | - |
| Captured CO_2_ (Q4) | MW | -157.29 | - |
| Methanation reactor (Q5) | MW | -313.09 | - |
| ICCU Reactor 2 (Q5) | MW | - | 196.87 |
| Product gas (Q6) | MW | -130.28 | -121.76 |
| Gas-liquid separator (Q7) | MW | -422.20 | -413.91 |
| Net energy input | MW | 933.93 | 196.87 |
| Net energy output | MW | 1681.58 | 995.78 |
| ∑ E_in_ ^a^ | MW | 2958.22 | 2221.16 |
| ∑ E_out_ ^b^ | Mwe | 1593.60 | 1351.51 |
| Energy efficiency (η) ^c^ | % | 53.98 | 60.94 |

^a)^ ∑E_in_ is the sum of the energy input to the coal-fired plant (2024.29 MW) and the net energy input to the CCU or ICCU.

^b)^ ∑E_out_ is the sum of the electricity production of a coal-fired power plant (1000 MWe) and the electricity production from waste heat recovery in the CCU or ICCU. (The efficiency of converting heat to electricity is 35.3% ^[10]^).

^c)^ The energy efficiency of the power plant with CCU or ICCU is defined as the ratio of total output to input energy. That is, η = ∑ E_out_ / ∑ E_in_ × 100%.

**Table S8.** Equipment cost estimation and parameters of the scaling function.

|  | **C_r_ ^a^ (M€)** | **Scaling parameter** | **S_r_ ^a^** | **f ^a^** | **C_m_ ^a^ (M€)** | | **Ref.** |
| --- | --- | --- | --- | --- | --- | --- | --- |
|  |  |  |  |  | **CCU** | **ICCU** |  |
| CO_2_ capture reactor | 353.70 | Heat duty, MW | 1027 | 0.98 | 189.80 | - | ^[16]^ |
| Calcination reactor | 13.60 | Outlet volume flow rate, m^3^/s | 838.6 | 0.98 | 5.72 | - | ^[16]^ |
| Methanation reactor | 3.80 | Outlet volume flow rate, t/h | 3.6 | 0.60 | 112.01 | - | ^[12]^ |
| ICCU reactor | 353.70 | Heat duty, MW | 1027 | 0.98 | - | 70.10 | ^[16]^ |
| Compressor | 0.49 | Net power, MW | 0.413 | 0.68 | 13.94 | - | ^[17]^ |
| Gas-liquid separator | 0.66 | Outlet volume flow rate, t/h | 6.59 | 0.73 | 20.26 | 20.26 | ^[17]^ |
| Steam cycles | 245.60 | Net power, MW | 568.5 | 0.69 | 193.41 | 133.69 | ^[16]^ |
| Total Equipment Cost (M€) |  |  |  |  | 535.14 | 224.05 |  |

^a)^ C_r_ represents the reference cost, S_r_ represents the reference size, and f represents the equipment scale factor index. The equipment cost C_m_ is obtained by the formula: $\text{C}_{\text{m}}\text{=}\text{ C}_{\text{r}}{\text{(}\frac{\text{S}}{\text{S}_{\text{r}}}\text{)}}^{\text{f}}$

**Table S9.** Summary of the economic evaluation of the CCU and ICCU.

|  | **CCU** | **ICCU** | **Assumption** |
| --- | --- | --- | --- |
| **Capital Costs** |  |  |  |
| Total Equipment Cost (M€) | 535.14 | 224.05 |  |
| Total install costs (M€) | 668.92 | 280.06 | 125% of the equipment cost |
| Process utilities and offsite unit costs (M€) | 133.78 | 56.01 | 25% of the equipment cost |
| Land, permitting, surveying, etc. (M€) | 33.45 | 14.00 | 5% of the installation cost |
| Owner's cost and contingency cost (M€) | 100.34 | 42.01 | 15% of the installation cost |
| Total Capital cost (M€) | 1471.63 | 616.14 |  |
| Annual capital cost (M€/year) | 162.17 | 67.90 |  |
| **O&M** |  |  |  |
| Plant lifetime (year) | 25 | 25 |  |
| Annual operational time (h) | 8000 | 8000 |  |
| Annual maintenance costs (M€/year) | 23.41 | 9.80 | 3.5% of the installation cost |
| Direct labor cost (M€/year) | 0.15 | 0.15 | 30 persons, 5000 €/person/year |
| Property taxes and insurance (M€/year) | 20.07 | 8.40 | 3% of the installation cost |
| Administrative, support and overhead cost (M€/year) | 0.05 | 0.05 | 35% direct labor cost |
| H_2_ (M€/year) | 1074.16 | 1074.16 |  |
| Adsorbents and catalysts (M€/year) | 50.57 | 31.25 |  |
| Coal (M€/year) | 297.49 | 62.71 |  |
| Total O&M cost (M€/year) | 1465.91 | 1186.52 |  |
| **TAC (M€/year)** | 1628.08 | 1254.42 |  |

^a)^ The exchange rate from the USD to the EUR is calculated as $/€=0.91.

^b)^ Costs estimated based on market prices.

**Table S10. Prices of objects involved in the process and carbon taxes in different regions.**

| **Materials** | **Specific cost (€/t)** | **Ref.** |
| --- | --- | --- |
| H_2_ | 1255.8 | ^[18]^ |
| CaO | 48.4 | estimated based on market prices |
| Ni/CeO_2_ | 2842.7 | estimated based on market prices |
| Coal | 160 | ^[12]^ |
| **Utility** | **Specific cost (€/MWh)** |  |
| **Electricity** | 98.3 | ^[19]^ |
| **Country** | **Carbon price (€/tCO_2_e)** |  |
| **China** | 8.2 | ^[20]^ |
| **USA** | 28.2 | ^[20]^ |
| **EU** | 79.2 | ^[20]^ |

^a)^ The exchange rate from the USD to the EUR is calculated as $/€=0.91.

## 4. Reference

[1] Z. Lv, T. Deng, C. Gao, Y. Zheng, C. Wu, J. Ran, C. Qin, *Chemical Engineering Journal* **2024**, *489*, 151427.

[2] H. Sun, Y. Zhang, C. Wang, M. A. Isaacs, A. I. Osman, Y. Wang, D. Rooney, Y. Wang, Z. Yan, C. M. A. Parlett, F. Wang, C. Wu, *Chemical Engineering Journal* **2022**, *437*, 135394.

[3] Z. Lv, J. Ruan, W. Tu, X. Hu, D. He, X. Huang, C. Qin, *Separation and Purification Technology* **2023**, *309*, 123044.

[4] J.-H. Woo, S. Jo, J.-E. Kim, T.-Y. Kim, H.-D. Son, H.-J. Ryu, B. Hwang, J.-C. Kim, S.-C. Lee, K. L. Gilliard-AbdulAziz, in *Catalysts, Vol. 13*, **2023**.

[5] H. Sun, Y. Wang, S. Xu, A. I. Osman, G. Stenning, J. Han, S. Sun, D. Rooney, P. T. Williams, F. Wang, C. Wu, *Fuel* **2021**, *286*, 119308.

[6] M. A. Arellano-Treviño, Z. He, M. C. Libby, R. J. Farrauto, *Journal of CO2 Utilization* **2019**, *31*, 143-151.

[7] M. A. Arellano-Treviño, N. Kanani, C. W. Jeong-Potter, R. J. Farrauto, *Chemical Engineering Journal* **2019**, *375*, 121953.

[8] S. B. Jo, J. H. Woo, J. H. Lee, T. Y. Kim, H. I. Kang, S. C. Lee, J. C. Kim, *Sustainable Energy & Fuels* **2020**, *4*, 4679-4687.

[9] A. Bermejo-López, B. Pereda-Ayo, J. A. González-Marcos, J. R. González-Velasco, *Journal of CO2 Utilization* **2019**, *34*, 576-587.

[10] Y. Qiao, W. Liu, R. Guo, S. Sun, S. Zhang, J. J. Bailey, M. Fang, C. Wu, *Fuel* **2023**, *332*, 125972.

[11] L. Zhu, Y. He, L. Li, P. Wu, *Energy* **2018**, *144*, 915-927.

[12] Z. Lv, H. Du, S. Xu, T. Deng, J. Ruan, C. Qin, *Applied Energy* **2024**, *355*, 122242.

[13] W. J. Lee, C. Li, H. Prajitno, J. Yoo, J. Patel, Y. Yang, S. Lim, *Catalysis Today* **2021**, *368*, 2-19.

[14] Y. Yang, R. Zhai, L. Duan, M. Kavosh, K. Patchigolla, J. E. Oakey, *International Journal of Greenhouse Gas Control* **2010**, *4*, 603-612.

[15] O. Y. H. Elsernagawy, A. Hoadley, J. Patel, T. Bhatelia, S. Lim, N. Haque, C. e. Li, *Journal of CO 2 Utilization* **2020**, *41*, 101280.

[16] M. Astolfi, E. De Lena, M. C. Romano, *International Journal of Greenhouse Gas Control* **2019**, *83*, 140-155.

[17] M. Marchese, G. Buffo, M. Santarelli, A. Lanzini, *Journal of CO2 Utilization* **2021**, *46*, 101487.

[18] Z. Xie, Z. Sun, B. Shao, Y. Zhu, R. Ma, S. Li, J. Li, Y. Chen, H. Liu, J. Hu, *Chemical Engineering Journal* **2024**, *495*, 153465.

[19] https://www,lazard.com/media/sptlfats/lazards-levelized-cost-of-energy-version-150-vfpdf.

[20] https://openknowledge.worldbank.org/entities/publication/a1abead2-de91-5992-bb7a-73d8aaaf767f.
